# A methotrexate labelled dual metal oxide nanocomposite for long-lasting anti-cancer theranostics

**DOI:** 10.1016/j.mtbio.2024.101377

**Published:** 2024-12-05

**Authors:** Joyce L.Y. Tang, Shehzahdi S. Moonshi, Yuao Wu, Gary Cowin, Karla X. Vazquez- Prada, Huong D.N. Tran, Andrew C. Bulmer, Hang Thu Ta

**Affiliations:** a Queensland Micro- and Nanotechnology Centre, Griffith University, Nathan, Queensland, 4111, Australia; b School of Environment and Science, Griffith University, Nathan, Queensland, 4111, Australia; c Australian Institute for Bioengineering and Nanotechnology, University of Queensland, St Lucia, Queensland, 4072, Australia; d National Imaging Facility, Centre for Advanced Imaging, University of Queensland, St Lucia, Queensland, 4072, Australia; e School of Pharmacy and Medical Sciences, Griffith University, Southport, Queensland, 4215, Australia

**Keywords:** Nanotheranostic, Chitosan, Cerium oxide, Nanoceria, Reactive oxygen species, Methotrexate, Cancer, Magnetic resonance imaging

## Abstract

We explored the feasibility of a self-assembled chitosan nanocomposite incorporating cerium oxide/nanoceria and superparamagnetic iron oxide nanoparticles (Chit−IOCO NPs), conjugated with methotrexate (MTX) and Cy5 dye, as an integrated cancer theranostic nanosystem (Chit-IOCO-MTX-Cy5). In this system, nanoceria serves as an anti-cancer agent, while the superparamagnetic iron oxide nanoparticles function as a negative contrast agent for MR imaging. This dual metal oxide nanocomposite is conjugated with MTX which is a structural analogue of folate, serving both as a targeting mechanism for folate receptors on cancer cells and as a chemotherapeutic drug. Chit−IOCO-MTX-Cy5 exhibited exceptional negative contrast in T2 and T2∗-weighted MRI, achieving a high relaxivity of 409.5 mM⁻^1^ s⁻^1^ which is superior to clinically approved agents. The nanocomposite demonstrated both pro-oxidative and antioxidative properties, significantly increasing reactive oxygen species (ROS) production in U87MG cells (1.4-fold change), which triggered apoptosis in these cancer cells. Simultaneously, it exhibited ROS scavenging activity in non-malignant endothelial cells (0.8-fold change). Intravenous infusion of Chit-IOCO-MTX-Cy5 (5 mg/kg MTX) led to significant tumor growth inhibition, indicating a synergistic enhancement of anti-cancer effects when combining MTX and nanoceria, compared to free MTX or nanoceria without MTX conjugation. Importantly, after treatment cessation, tumours in the nanocomposite group did not re-grow, while those in the free MTX group rapidly did. *In vivo* MR and fluorescence imaging revealed improved uptake and retention of Chit−IOCO-MTX-Cy5 in tumours compared to nanoceria without MTX. Notably, biosafety and biochemical analyses in mice showed no significant differences between the Chit−IOCO-MTX-Cy5 treatment group and control groups.

## Graphical abstract

**TOC Figure:** Nanohybrid platform of chitosan nanocomposites comprising cerium oxide and superparamagnetic iron oxide NPs (Chit−IOCO) conjugated to methotrexate (MTX) as an integrated cancer theranostic nanosystem. *In vivo* MRI displayed enhanced uptake of targeted MTX conjugated NPs in tumours indicating selectivity of the nano system which resulted in significant synergistic reduction in tumour mass.

**Figure:**
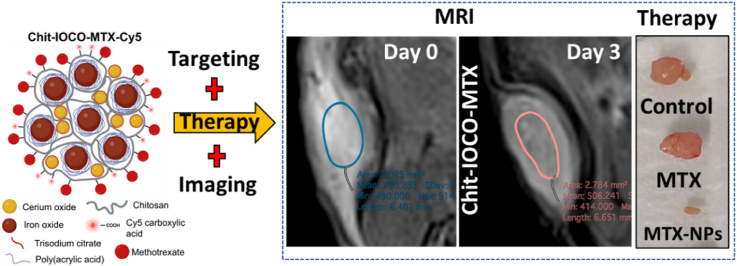

## 1 Introduction

Cancer is among the leading causes of death worldwide, with numbers expected to increase in the oncoming years [1]. One of the current approaches to managing cancer employs one or more diagnostic imaging modalities such as magnetic resonance imaging (MRI) to confirm cancer phenotype and heterogeneity prior to treatment which can involve the administration of systemic chemotherapy with cytotoxic drugs, or directed radiation therapy [2]. However, a major drawback of conventional approaches is the administration of different materials as imaging contrast and chemotherapeutic agents in separate occasions, leading to differences in biodistribution and selectivity to the target disease sites [3,4]. Potent and repeated doses are usually required to overcome low bioavailability of drugs reaching the disease sites and unfavourable biodistribution of drugs at non-target sites. Consequently, these unfavourable outcomes will have a detrimental effect on the survival and growth of healthy cells and develop susceptibility to multidrug resistance (MDR) [5,6]. To overcome these drawbacks, theranostics employs the use of nanomaterials combining controlled and targeted drug delivery with simultaneous diagnosis, treatment, and real time monitoring of therapy response in a single integrated system [7,8]. However, clinical translation remains challenging due to the low number of clinical trials that have tested theranostic approaches to cancer diagnosis and treatment. Furthermore, no nanotheranostic material are currently approved by the United States Food and Drug Administration (FDA) despite the high number of successful pre-clinical studies [9].

Cancer cells adapting to tumour microenvironment (TMEs) have increased basal ROS level with a higher rate of both ROS production and ROS scavenging in comparison to healthy cells, which potentially increase their susceptibility to ROS or redox manipulation therapies [10]. Thus, enhancing ROS production and/or inhibiting ROS elimination to force excessive accumulation in tumours and trigger oxidative stress-induced cancer cell death are promising strategies as effective cancer therapies [[11], [12], [13], [14]]. Cerium oxide nanozymes or nanoceria have presented itself as a potential anti-cancer agent based on its unique chemistry of cerium oxide that allow both pro-oxidant and antioxidant activities manipulating ROS levels in cancer cells [[15], [16], [17], [18], [19]]. This capability permits nanoceria to exhibit either antioxidant or pro-oxidant characteristics [[20], [21], [22], [23]]. The oxidation states of nanoceria displayed pH sensitivity with higher efficacy of ROS generation in more acidic environment (pH 5.4 > pH 6.5 > pH 7.4^24^) and vice versa for ROS scavenging [[25], [26], [27]]. Thus, the variation in acid-base status between normal and tumour tissues can be exploited and is instrumental in anticancer drug design and discovery [28,29]. Therefore, cerium oxide NPs have been developed to exert anticancer effects by triggering oxidative stress resulting in apoptosis in cancer cells while protecting normal tissues by attenuating free radicals.

MRI in combination with other imaging modalities, are employed to detect and monitor primary and metastatic tumours [[30], [31], [32]]. For better visualisation and diagnosis purposes, MRI requires the use of contrast agents to enhance signal-to-noise (SNR) and contrast-to-noise (CNR) ratios. Superparamagnetic iron oxide nanoparticles (SPIONs) have attracted increasing interests as an MRI contrast agent (negative contrast in T_2_/T_2_∗ weighted MR images) advantaging from the small size, superparamagnetic properties, relatively lower toxicity, biodegradability, surface functionalisation and/or modification versatility and ability to integrate cancer diagnosis and therapy [[33], [34], [35], [36], [37]].

Research focus in recent years has shifted to simplifying and advancing the nanoplatform that contains molecules potentially displaying dual- or even multi-functionality such as dual-acting molecules that can be exploited as both therapeutic agent [38] and targeting ligand [39]. Chemotherapeutic drug methotrexate (MTX) being the synthetic analogue of folic acid has thus attracted interests in cancer theranostics as dual-acting ligand for active targeting and efficient cancer treatment [40]. MTX inhibits tumour growth by the indirect inhibition of rapid cell proliferation through blockage of folate-dependent enzymes that are essential in DNA production and cell proliferation. Hence, to target folate transporters, NPs can either be functionalised with folic acid or alternatively with folic acid analogues like MTX that employ the same FR mediated endocytic pathway and intracellular folate-dependent metabolic pathways [41]. Therefore, loading of MTX into functionalised nanocarriers is introduced as a viable option that can prolong plasma half-life, enhance drug accumulation at tumour sites, improve drug efficacy and allow controlled drug release.

We recently synthesised Chit−IOCO nanosystem comprising of SPIONs and nanoceria encapsulated in chitosan nanocarrier, employing nanoceria as ROS scavenging therapeutic module for theranostic of inflammatory diseases such as atherosclerosis and hepatitis [42,43]. The Chit−IOCO nanocomposites demonstrated effective *anti*-ROS capabilities by reducing ROS level of lipopolysaccharide-stimulated macrophages to basal level. The theranostic nanocomposites also showed effective MRI contrast efficacy with strong T_2_-weighted MR contrast and high MRI relaxitivity [42]. Here, the potential applications of this dual metal oxide nanocomposite are then extended further to theranostics of cancers, incorporating chemotherapeutic antifolate MTX to enhance cancer killing and for active cancer targeting. This work investigated the feasibility of a chitosan nanocomposites containing cerium oxide and superparamagnetic iron oxide NPs (Chit−IOCO) conjugated to methotrexate (MTX) as an integrated cancer theranostic nanosystem.

In this study, cerium oxide (nanoceria) was employed as an anticancer module due to its unique chemistry, which enables it to exhibit both pro-oxidant and antioxidant activities [15,16]. This capability allows for the manipulation of ROS levels in cancer cells, inducing oxidative stress that leads to apoptosis while simultaneously protecting normal tissues by reducing free radicals. Superparamagnetic iron oxide [[44], [45], [46], [47], [48], [49]] NPs were utilised as negative contrast agent, enabling MR imaging to track the delivery of our NPs to the target site. MR imaging allows us to visualise the biodistribution and accumulation of NPs within tumours or organs *in vivo*. Hence, in this study, we employed IONPs as a negative contrast agent for MR imaging presenting it as a theranostic nanosystem which includes both the therapeutic (cerium oxide - CO) and imaging element (iron oxide - IO) in 1 platform.

Importantly, the nanocomposites were functionalised with the chemotherapeutic agent MTX, serving both as a targeting mechanism for folate receptors on cancer cells and as an anti-cancer drug. Our primary objective was to utilize methotrexate (MTX) as an active targeting ligand due to its structural similarity to folate [50]. Folate receptors are overexpressed in many cancer cells, making them ideal targets for targeted therapies. By conjugating MTX to our nanocarrier system, we aimed to enhance the selective delivery of therapeutic agents directly to these cancer cells. This approach leverages the mechanism of folate receptor-mediated endocytosis, where cells internalize folate and its analogues through specific receptor interactions. In cancers with high folate receptor expression, the conjugation of MTX not only facilitates improved binding to the target cells but also enhances the internalization of the therapeutic payload [51]. This can lead to a more effective treatment by increasing the concentration of the drug within the cancer cells while minimizing exposure to surrounding healthy tissues. Furthermore, using MTX in this way could potentially exploit the existing cellular mechanisms that tumours use to uptake folate, thereby improving the therapeutic efficacy of the treatment and reducing side effects commonly associated with traditional chemotherapy [51]. Overall, our strategy aims to improve the precision and effectiveness of cancer therapy through targeted drug delivery.

**Scheme 1:**
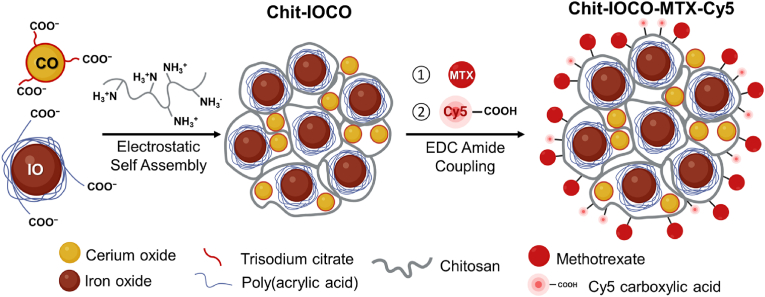
Schematic diagram illustrating synthesis of Chit−IOCO−MTX with Cy5 as the fluorescent reporter (not drawn to scale). Chit−IOCO nanoparticles were prepared through the electrostatic self-assembly between the positively charged chitosan and negatively charged trisodium citrate coated cerium oxide (CO−TSC) and poly(acrylic acid) coated iron oxide (IO−PAA) nanoparticles. Methotrexate (MTX) and Cy5 with intrinsic carboxylic groups −COOH were conjugated to the amino groups −NH_2_ of Chit-IOCO through EDC amide coupling reaction.

MTX despite being a potent cytotoxic drug and widely prescribed for a broad spectrum of diseases, is known to have narrow a therapeutic window owing to its short plasma half-life, poor aqueous solubility, and low permeability leading to near-lethal doses required for effective cancer killing [52]. On the other hand, nanoceria has been widely investigated as antioxidant in treating ROS-related diseases through ROS reduction whilst its pro-oxidant activity for therapeutic applications is less studied. Studies in the anti-cancer potential of nanoceria only emerged in recent years exploring its pro-oxidant properties inducing cancer cell death whilst the appealing antioxidant properties selectively shield normal cells and tissues from ROS, cellular damage, and cell death [16,53]. This nanocomposite is specifically designed for effective tracking of material delivery to targeted sites. Thus, exploiting the therapeutic capabilities of nanoceria and combining anticancer activities of nanoceria and MTX for synergistic treatment response could possibly allow lower dosage of MTX required to achieve effective cancer treatment while decreasing adverse effects. The drawbacks coming from the physicochemical properties of MTX could also be resolved as the MTX will be delivered through a biocompatible nanosystem that improves blood circulation, water solubility and permeability. Additionally, the synthesis of the Chit-IOCO nanocomposite employs electrostatic self-assembly, a method chosen for its simplicity and cost-effectiveness which reduces the need for complex machinery and harmful chemicals, scalability, and reproducibility which is crucial for commercialization and clinical application [54]. This combination of therapeutic agents, imaging agent and targeting moieties in a single nanoplatform hypothetically allows simultaneous imaging of tumour sites, targeted delivery of anticancer drugs to cancer cells, real-time tracking of biodistribution and monitoring of treatment response *in vivo*. These Chit−IOCO−MTX NPs were labelled with Cy5 as the fluorescent reporter (Scheme 1). We compared the effects of free MTX, Chit-IOCO (the nanocomposite), and Chit-IOCO-MTX (the targeted formulation) to evaluate differences in therapeutic outcomes both *in vitro* and *in vivo* in a U87-MG mouse model.

## 2 Methods

### 2.1 Materials

All chemicals were reagent grade and utilised without further purification unless specified. Polyacrylic acid (PAA), iron(III) chloride hexahydrate (FeCl_3_ · 6H_2_O), ammonium iron(II) sulphate hexahydrate ((NH_4_)_2_Fe(SO_4_)_2_ · 6H_2_O), ammonium cerium nitrate ((NH_4_)_2_Ce(NO_3_)_6_), sodium acetate trihydrate (CH_3_COONa · 3H_2_O), glacial acetic acid, low molecular weight chitosan, methotrexate hydrate, 1-ethyl-3-(3-dimethylaminopropyl)carbodiimide (EDC), MES hydrate and paraformaldehyde (PFA) powder were purchased from Sigma-Aldrich. Ammonia solution (30 % ammonia hydroxide in H_2_O) and trisodium citrate (TSC) were purchased from Chem-supply. Cyanine5 carboxylic acid (Cy5−COOH) was purchased from Lumiprobe. 70 % nitric acid was purchased from Ajax Finechem. High and low glucose formulated Dulbecco's Modified Eagle Medium (DMEM), Roswell Park Memorial Institute (RPMI) 1640 medium, fetal bovine serum (FBS) with and without heat inactivation, penicillin-streptomycin, Dulbecco's phosphate-buffered saline (DPBS) without calcium and magnesium, TrypLE™ Express Enzyme were purchased from Gibco™ (Thermo Fisher Scientific). PrestoBlue™ was purchased from Invitrogen™ (Thermo Fisher Scientific). 2′, 7′-dichlorofluorescin diacetate (DCFDA), Triton X-100, low gelling temperature agarose, Corning® Matrigel® Basement Membrane Matrix were purchased from Sigma-Aldrich. Water purified using a Millipore Milli-Q lab water system was used in all experiments. Sodium chloride 0.9 % for injection BP was purchased from Pfizer.

### 2.2 Synthesis of Chit−IOCO

Iron oxide, cerium oxide NPs and chitosan nanocomposites were synthesised as reported in our previous studies [21,42,55] and as below.

#### 2.2.1 Synthesis of iron oxide (IO−PAA)

Poly(acrylic acid) coated iron oxide NPs (IO−PAA) were prepared by co-precipitation method [21]. In brief, PAA (0.8 g, 0.44 mmol) was first dissolved in 200 mL of Milli-Q water. The PAA solution was purged with nitrogen for 30 min to remove oxygen and heated to reflux in an oil bath at 130 °C. FeCl_3_ · 6H_2_O (0.5512 g, 2.04 mmol) and (NH_4_)_2_Fe(SO_4_)_2_ · 6H_2_O (0.396 g, 1.01 mmol) were combined and dissolved in 4 mL of 37 % HCl, then added quickly into the hot PAA solution and allowed to stir for 5 min. 60 mL of ammonia solution was added into the mixture, and the mixture was refluxed for 2 h. The resulting solution was centrifuged at 2000 g for 5 min to remove large agglomerates. The supernatant consisted of NPs was concentrated using a 50 kDa molecular weight cut-off Amicon® centrifugal filter (Millipore, Inc.). The concentrated solution was dialysed against 5 L of Milli-Q water at pH 7 for one day. The dialysed IO−PAA was collected, concentrated as above, and stored at 4 °C.

#### 2.2.2 Synthesis of cerium oxide (CO−TSC)

Cerium oxide (CO) NPs were prepared by co-precipitation method [42]. (NH_4_)_2_Ce(NO_3_)_6_ (2.74 g, 5 mmol) and CH_3_COONa · 3H_2_O (10 g, 73.49 mmol) were dissolved in 70 mL Milli-Q water and 10 mL glacial acetic acid was added to the solution. The mixture was stirred at room temperature for 1 h then heated at 100 °C using an oil bath with condensation reflux for 2 h. The resulting yellow precipitates were collected by centrifuging at 6000 g for 10 min and washing twice with Milli-Q water. The precipitated CO NPs was then resuspended in 20 mL of Milli-Q water. To prepare trisodium citrate coated cerium oxide NPs (CO−TSC), 0.1 g of CO NPs was mixed with 15 mL of 0.1 M TSC in water for 24 h. The mixture was then filtered using 100 k molecular weight cut-off Amicon® centrifugal filter units (Millipore, Inc.) at 16,000 g for 1 min to remove large agglomerates. The resulting CO−TSC NPs were stored in 0.1 M TSC solution at 4 °C and dialysed against Milli-Q water using 10 k molecular weight cut-off Snakeskin tube (Thermo Scientific™) immediately before use.

#### 2.2.3 Synthesis of chitosan/dual metal oxide nanocomposite (Chit−IOCO)

Chit−IOCO nanocomposites were prepared via ionic gelation method [42,55] which is the self-assembly between positively charged chitosan and negatively charged IO−PAA and CO−TSC NPs. Chitosan was prepared by dissolving 1 g of chitosan in 1 L deionised water with 1 mL glacial acetic acid. The chitosan solution was filtered through 0.45 μm membrane and adjusted pH to 4.8. To synthesise Chit−IOCO, CO−TSC was first dialysed against 1 L Milli-Q for 15 min. Then, using a syringe pump programmed at 0.4 mL/min, a 1 mL mixture of IO−PAA (6 mg Fe) and CO−TSC (6 mg Ce) was pumped into 9 mL of chitosan solution. The mixture was incubated at room temperature for 1 h and centrifuged at 11,000 g, 4 °C for 30 min. The pellet was dispersed into 1 mL pH 4.8 water (deionised water with pH adjusted to 4.8 with acetic acid) through sonication at 60 % amplitude for 4.5 min (30 s ON/30 s OFF) on ice.

### 2.3 Conjugation of methotrexate (MTX) and fluorescent dye to Chit−IOCO

#### 2.3.1 Synthesis of Chit−IOCO−Cy5 and Chit−IOCO−MTX−Cy5

MTX and fluorescent dye Cy5 were conjugated to Chit−IOCO via the formation of amide bonds between chitosan's amino groups (−NH_2_) and MTX's and/or Cy5's carboxylic groups (−COOH) in the presence of EDC that activates −COOH functional groups. A 9 mL mixture of Chit−IOCO (11.96 g, 4.14 μmol), EDC (0.85 mg, 4.43 μmol) and MTX (1.35 mg, 2.98 μmol) in pH 4.8 water was first reacted for 2 h at room temperature. Cy5−COOH (0.051 mg, 0.098 μmol) was pre-activated with EDC (0.188 mg, 0.98 μmol) in 1 mL 0.1 M, pH 5 MES buffer for 15 min on a shaker, added to the mixture and stirred overnight at room temperature. The resulting solution was centrifuged at 11,000 g, 4 °C for 30 min. The pellet was dispersed into 1 mL pH 4.8 water and sonicated at 30 % amplitude for 2 min (20 s ON/20 s OFF) on ice. Different mole ratios of reactants, choices of solvents, incubation times, conditions of carboxylic groups activation by EDC and parameters of sonication were used to optimise the synthesis of Chit−IOCO−MTX−Cy5.

#### 2.3.2 Determination of MTX and Cy5 conjugation

To determine the conjugation ratio of MTX, the absorbance of the supernatant from Chit−IOCO−MTX−Cy5 synthesis was measured at 300 nm with the supernatant from Chit−IOCO−Cy5 synthesis used as blank correction. The amount of unconjugated MTX was calculated based on the respective calibration curve. The encapsulation efficiency (EE %) of conjugated MTX and Cy5 were calculated using the following equations:

$$Cy5\;EE\;\left(wt\;\%\right)=\frac{weight\;of\;Cy5\;in\;NPs}{weight\;of\;Cy5\;fed}\times 100$$

$$MTX\;EE\;\left(wt\;\%\right)=\frac{weight\;of\;MTX\;fed-weight\;of\;MTX\;in\;supernatant}{weight\;of\;MTX\;fed}\times 100$$

### 2.4 Characterisation of NPs

The average size, size distribution and zeta potential of NPs were measured using the Litesizer™ 500 (Anton-Paar). Transmission Electron Microscopy (TEM) images were collected on a JEM-1010 Transmission Electron Microscope (JEOL Ltd.) at Australian Institute for Bioengineering and Nanotechnology (AIBN), University of Queensland (St Lucia, Brisbane) operating at an accelerating voltage of 120 kV. The samples on the copper grid were stained with phosphotungstic acid (10 mg/mL, pH adjusted to 7.3 by sodium hydroxide) for 30 s to visualise chitosan and the remaining acid solution was removed gently by a piece of tissue. The iron (Fe) and cerium (Ce) concentrations of NPs were determined via Inductively Coupled Plasma-Optical Emission Spectroscopy (ICP-OES) and -Mass Spectrometry (ICP-MS) respectively. NPs were dissolved in 7 % HNO_3_ and submitted to ALS Geochemistry (Stafford, Brisbane) for ICP testing.

### 2.5 Phantom magnetic resonance imaging (MRI)

The MRI scans and T_2_ relaxation time of the Chit−IOCO−MTX−Cy5 NPs at concentrations equivalent to 3.6, 7.2, 14.4 and 28.8 μg/mL Fe (equivalent to 0.06, 0.13, 0.26 and 0.52 mM Fe), were acquired using a Bruker 9.4 T MRI scanner running Paravision 6.0.1 hosted by the Centre of Advanced Imaging (CAI), University of Queensland (St Lucia, Brisbane). T_2_ values were calculated using a 2D multislice multi echo spin echo sequence using TR = 2630 ms, TE = 10 ms, 32 echoes, flip angle of 30°, 0.117 × 0.117 mm in-plane resolution. The acquisition time was 11 min 30 s. The T_2_-weighted relaxivity (*r*_*2*_) is defined as the slope of the linear regression plotted from the measured relaxation rate (1/T_2_) versus the concentration of the contrast agent Fe.

### 2.6 *In vitro* study

#### 2.6.1 Cell culture

Cancer cells U-87 MG, HCT 116, B16-F10, SK-OV-3, MCF7 and MDA-MB-231 and endothelial cells SVEC4-10 were attained from the American Type Culture Collection. U-87 MG cells were maintained in DMEM low glucose (1 g/L), B16-F10 and SVEC4-10 cells in DMEM high glucose (4.5 g/L) and the remaining cell lines in RPMI 1640 medium, at 37 °C in a humidified atmosphere with 5 % CO_2_ [56]. All growth media were supplemented with 10 % fetal bovine serum (FBS), 100 U/mL penicillin and 100 U/mL streptomycin, with the exception of SVEC4-10 cells supplemented with 10 % heat-inactivated FBS. Cells were passaged or seeded for experiments at approximately 90 % confluency.

#### 2.6.2 Cytotoxicity assay

U-87 MG, HCT 116, B16-F10, SK-OV-3, MCF7, MDA-MB-231 and SVEC4-10 cells were seeded into 96-well plates at cell densities of 10,000 cells/well and allowed to attach overnight. Cells were treated with different concentrations of free MTX, Chit−IOCO and Chit−IOCO−MTX for 48 h. After treatment, cells were washed with PBS and incubated with 1 × PrestoBlue® cell viability reagent for 30 min at 37 °C and 5 % CO_2_. Viable cells were detected by fluorescence intensity measurement using CLARIOstar® *Plus* plate reader (BMG Labtech) at 560/590 nm (excitation/emission). The fluorescence of non-treated cells was determined as 100 % cell viability. Sigmoidal dose-response curves and half maximal inhibitory concentration (IC_50_) values were produced from non-linear regression analyses using GraphPad Prism. Combination index (CI) was calculated using the formula below [57],

$$CI=\frac{\text{NP}\left[\text{MTX}\right]}{\left[\text{MTX}\right]}+\frac{\text{NP}\left[\text{Ce}\right]}{\left[\text{Ce}\right]}$$

where NP[MTX] and NP[Ce] represented the IC_50_ values of Chit−IOCO−MTX in terms of MTX and Ce concentrations, and [MTX] and [Ce] represented the IC_50_ values of free MTX and Chit−IOCO in terms of MTX and Ce concentration respectively.

#### 2.6.3 Intracellular ROS assay

U-87 MG, HCT 116 and SVEC4-10 cells were seeded in 96-well plates at a density of 10,000 cells/well and allowed to attach overnight. To assess the variations in basal ROS levels of different cell lines, non-treated cells were allowed to proliferate for 48 h, washed with DPBS and stained with 25 μM DCFDA reagent for 45 min at 37 °C and 5 % CO_2_. To determine the ROS regulating effects of NPs, cells were first treated with free MTX, Chit−IOCO or Chit−IOCO−MTX in growth media for 8 h, washed with DPBS and stained with DCFDA reagent as above. Intracellular ROS levels were measured by taking fluorescence readings at 485/535 nm (excitation/emission) using CLARIOstar® *Plus* plate reader (BMG Labtech). The fluorescence intensities of treated wells were normalised against non-treated wells to determine the fold change of intracellular ROS level.

#### 2.6.4 Cell apoptosis assay

Annexin V-FITC assay was utilised to assess apoptosis of U87-MG cells (1 × 10^5^ cells/well) in 24 well plates. After 24 h, cells were treated with free MTX, Chit−IOCO or Chit−IOCO−MTX NPs for 24 h. All attached and floating cells were collected and washed thrice with PBS. Cells were suspended in binding buffer, stained with Annexin V-FITC and propidium iodide (PI) for 15 min in the dark. Stained cells were analysed using the Accuri6 cytometer and Flowjo software.

#### 2.6.5 *In vitro* cell uptake of NPs

U-87 MG cells were seeded in a 6-well plate at a density of 500,000 cells/well and allowed to attach overnight. Cells were treated with Chit−IOCO−Cy5 and Chit−IOCO−MTX−Cy5 at concentrations equivalent to 1.8, 3.6, 7.2, 14.4 and 28.8 μg/mL Fe for 8 h. Subsequently, cells were washed with DPBS for 3 times, detached using TrypLE™ Express and centrifuged at 200 g for 5 min. The cell pellets were resuspended with 200 μL of warm 1 % low-gelling temperature agarose. 50 μL of cells in agarose was quickly transferred into a prepared phantom vessel and allowed to solidify at room temperature. The phantoms were imaged on Sapphire™ Biomolecular Imager (Azure Biosystems) with a 658 nm excitation laser coupled to a 670–750 nm filter. Fluorescence images were processed using Fiji software [58]. T_2_*∗* values were determined using 2D multigradient echo (MGE) sequence with TR = 300 ms, TE = 3 ms, 16 echoes, 0.117 × 0.117 mm in-plane resolution. The acquisition time was 5 min 7 s.

#### 2.6.6 Haemolysis assay

Fresh blood was collected from laboratory members voluntarily and centrifuged at 1000 rpm for 15 min with soft acceleration and break (set at 4). The supernatant (plasma) was removed, and the red blood cells (RBCs) were washed twice with PBS (pH 7.4) at 1000 rpm for 15 min. 1 mL of the RBCs was diluted with PBS (pH 7.4) to create a 50 mL RBCs stock. 20 μL of sample (Chit−IOCO−Cy5 and Chit−IOCO−MTX−Cy5 with Ce concentration of 0.125, 1.25, 12.5 and 125 μg/mL) was added to 180 μL of RBCs stock solution and placed on a 37 °C shaking incubator for 1 h 1 % Triton-X 100 and pH 7.4 DPBS were used as positive and negative haemolysis controls respectively. The samples were then centrifuged at 14,000 g for 15 min. The supernatant was collected for absorbance measurement at 545 nm using CLARIOstar® *Plus* (BMG Labtech) plate reader. The absorbance of 1 % Triton-X 100 treated sample was determined as 100 % haemolysis.

### 2.7 *In vivo* study

#### 2.7.1 Animal study

Animal studies were conducted at the Centre of Advanced Imaging (CAI), University of Queensland (St Lucia, Brisbane), in accordance with the national guidelines provided and approved by the institutional animal care and ethics committees of the University of Queensland (# 2021/AE001086). All animals were sourced from Animal Resources Centre (Murdoch, Perth) and acclimatised for full seven days at CAI animal holding room prior to commencement of experiments. Procedures including tumour induction, treatment administration, fluorescence and MR imaging and terminal blood collection were performed on mice placed under isoflurane anaesthesia (1–4 % isoflurane with an air-oxygen mixture at a flow rate of 1 L/min). Throughout the experiment period, animals were monitored for signs of distress or ill health with appropriate husbandry and supportive therapies including deep and clean supportive bedding, warm, quiet and dim environment, fluids and food and water in a palatable readily available form provided.

#### 2.7.2 Biosafety assessment

10-week-old female C57BL/6J mice were randomly divided into three groups and administered with saline (*n* = 5), Chit−IOCO−MTX−Cy5 (5 mg/kg MTX, *n* = 5) or Chit−IOCO−MTX−Cy5 (2.5 mg/kg MTX, *n* = 5) via intravenous (IV) tail vein injection to assess the biocompatibility of NPs. The treatment administration took place on days 0, 4 and 8. The mice were monitored every 3 days for changes in body weight. 5 days after final injection, mice were placed under deep anaesthesia and 600–800 μL of blood were collected from each mouse by cardiac puncture. The whole blood was left undisturbed at room temperature for 30 min to allow clotting, then centrifuged at 2000 g for 10 min at 4 °C. The resulting supernatant (serum) was collected immediately and stored at −20 °C for further testing. Clinical chemistry analysis of serum (glucose, bilirubin, albumin, alanine transaminase, creatinine, urea and uric acid) was completed on a Beckman Coulter AU480 analyser (Lane Cove, Australia). Frozen aliquots were thawed, centrifuged at 21,500 g for 10 min (room temperature) prior to analysis. All analyses were conducted after the instrument passed calibration (system check) and quality control. Samples were analysed in single or duplicate, with duplicate measures averaged. Data were compared to reference ranges for rodents (rats) as per published results [59]. After blood collection, major organs including heart, lung, liver, spleen and kidney were harvested for weighing and then stored in 4 % PFA solution, sliced and stained with hematoxylin and eosin (H&E) for histological analysis.

#### 2.7.3 Therapeutic efficacy

To establish tumour models, 10-week-old mixed gender BALB/c nude mice were each subcutaneously injected with 100 μL U-87 MG cells (7.5 × 10^6^ cells suspended in 1:1 ratio of Matrigel® Basement Membrane Matrix and L-15 medium) into the flank. The tumour size was monitored twice a week using a digital vernier calliper, and the tumour volume (*V*) was calculated using the formula: *V* = (Length × Width [2])/2. When tumour volume reached 100 mm^3^, mice were randomly divided into six groups (*n* = 4) and intravenously injected through tail vein with 100 μL saline, free MTX (5 mg/kg), Chit−IOCO−Cy5 (0.7 mg/kg Ce), Chit−IOCO−Cy5 (0.35 mg/kg Ce), Chit−IOCO−MTX−Cy5 (5 mg/kg MTX, 0.7 mg/kg Ce) and Chit−IOCO−MTX−Cy5 (2.5 mg/kg MTX, 0.35 mg/kg Ce). The treatment administration took place on days 0, 4 and 8. The anti-cancer efficacies of the treatment groups were assessed by monitoring tumour volume and body weight twice a week. At the end of the experiment, mice were sacrificed, and tumours were weighed and harvested. TUNEL assay of tumours was performed using a kit (ab206386) as per manufacturer's instructions.

#### 2.7.4 Targeting efficacy

The biodistribution of the NPs was monitored real-time using an *in vivo* fluorescence imaging system IVIS Lumina X5. Whole-body imaging was performed on anaesthetised U-87 MG tumour-bearing mice at 4 and 24 h post tail vein injection with Chit−IOCO−Cy5 (0.7 mg/kg Ce) and Chit−IOCO−MTX−Cy5 (5 mg/kg MTX, 0.7 mg/kg Ce). Fluorescence intensities were determined using the ROI function of Aura Imaging Software (Spectral Instruments Imaging).

#### 2.7.5 Magnetic resonance imaging (MRI) efficacy

Anaesthetised mice were placed in a 300 mm bore 7 T ClinScan preclinical MR Scanner running Siemens VB17 (Bruker/Siemens). A mouse body radiofrequency coil with an inner diameter = 40 mm was used to acquire MR images at 0-, 1-, 3-day post first IV injection and 5-day post second IV injection with Chit−IOCO−Cy5 (1.4 mg/kg Fe) and Chit−IOCO−MTX−Cy5 (1.2 mg/kg Fe). 2D T_2_ spin echo images were acquired using the following parameters: TR = 1200 ms, TE = 12.7, 25.4, 38.1, 50.8, 63.5 ms, field of view 30 × 30 mm, slice thickness = 1 mm, number of slices = 26, slice gap = 1.2 mm, image matrix = 192 × 192, flip angle = 180°, pixel bandwidth = 130 Hz/Px, total scan time was 3 min 50 s. MRI images acquired were processed using the Horos software (Horos Project).

### 2.8 Statistical analysis

Data are presented as mean ± standard deviation unless otherwise stated. One-way ANOVA (with Tukey's and Dunnett's multiple comparison tests as *post hoc*) and student t-test were employed for significance testing with *p* value ≤ 0.05 considered statistically significant. Data analyses were performed using GraphPad Prism (GraphPad Software Inc.).

## 3 Results

### 3.1 Synthesis and characterisation of IO−PAA, CO−TSC, Chit−IOCO, Chit−IOCO-Cy5 and Chit−IOCO-MTX-Cy5

**Fig. 1:**
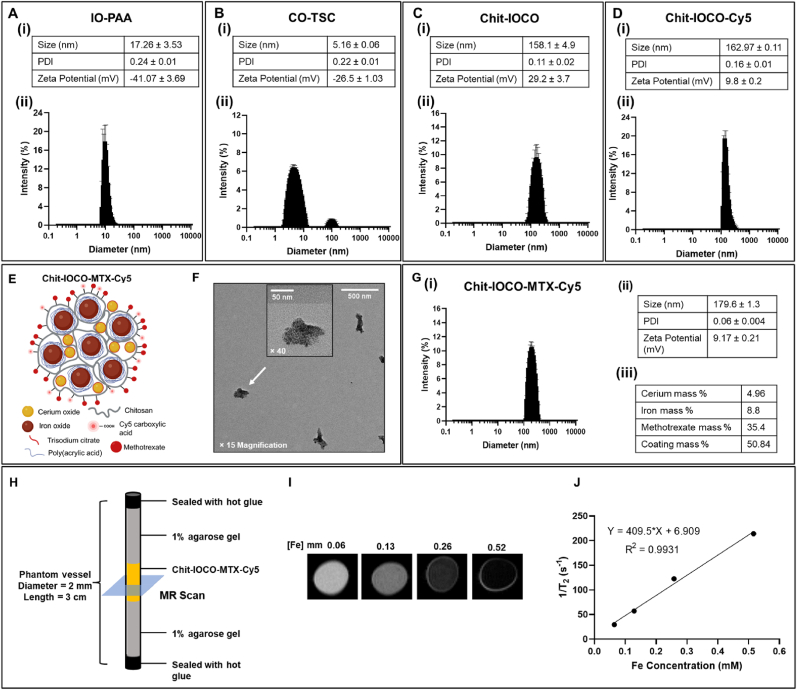
Characterisation of IO−PAA, CO−TSC, Chit−IOCO, Chit-IOCO-Cy5 and Chit-IOCO-MTX-Cy5 nanoparticles. (A) (i) Nanoparticle size, polydispersity index (PDI) and zeta potential of IO−PAA. (ii) Dynamic light scattering size distribution of IO−PAA nanoparticles. (B) (i) Nanoparticle size, PDI and zeta potential of CO−TSC. (ii) Dynamic light scattering size distribution of CO−TSC nanoparticles. (C) (i) Nanoparticle size, PDI and zeta potential of Chit−IOCO. (ii) Dynamic light scattering size distribution of Chit−IOCO nanoparticles. (D) (i) Nanoparticle size, PDI and zeta potential of Chit−IOCO-Cy5. (ii) Dynamic light scattering size distribution of Chit−IOCO-Cy5 nanoparticles. (E) Illustrated structure of Chit−IOCO−MTX-Cy5 (not drawn to scale). (F) TEM image of Chit−IOCO−MTX−Cy5 at × 15 and × 40 magnifications. (G) (i) Dynamic light scattering size distribution of Chit−IOCO-MTX-Cy5 nanoparticles. (ii) Nanoparticle size, PDI and zeta potential of Chit−IOCO-MTX-Cy5. (iii) Mass percentage of NP components. MRI of Chit−IOCO−MTX−Cy5 phantoms. (H) Scheme of MR phantom preparation and MR scan position. (I) MRI images of Chit−IOCO−MTX−Cy5 phantoms. Chit−IOCO−MTX−Cy5 nanoparticles were diluted to different concentrations of iron (Fe), sealed in phantom vessels as illustrated in (H), and imaged using a 9.4 T MRI. (J) Relaxation rate (1/T_2_) plotted against Fe concentrations of Chit−IOCO−MTX−Cy5 using relaxation time (T_2_) automatically generated from T_2_-weighted scans by the operating system.

IO−PAA and CO−TSC NPs were both successfully synthesised using coprecipitation method. The hydrodynamic size of IO−PAA was 17.26 nm with a polydispersity index (PDI) of 0.24. The IO−PAA was negatively charged with a zeta potential of −41.07 mV due to the PAA coating (Fig. 1A). CO−TSC had a hydrodynamic size of 5.16 nm with a PDI of 0.22. The CO−TSC was also negatively charged (−26.5 mV) due to the TSC coating (Fig. 1B). Chitosan nanocomposites containing iron oxide and cerium oxide were prepared by the electrostatic self-assembly method through the controlled addition of negatively charged IO−PAA and CO−TSC to positively charged chitosan solution. The resulting Chit−IOCO had an average hydrodynamic size of 158.1 nm, a low PDI of 0.11 and was positively charged at 29.2 mV (Fig. 1Ci). The dynamic light scattering plot revealed narrow size distribution of Chit−IOCO (Fig. 1Cii).

The synthesised Chit−IOCO went through further modifications via loading of anti-cancer agent and fluorescent dyes. The double amount of MTX were added as an effort to achieve higher concentration of MTX in the NPs as MTX is generally administered at high dosage for cancer treatment [60]. Different mole ratios of reactants, choices of solvents, incubation times, conditions of carboxylic groups activation by EDC and parameters of sonication were used to optimise the synthesis of Chit−IOCO-MTX−Cy5. In summary (Figs. S1 and S2), Chit−IOCO−MTX−Cy5 was successfully synthesised in a multi-step reaction started with 2 h reaction of MTX, EDC and Chit−IOCO, followed by 15 min pre-activation of Cy5−COOH with EDC in 0.1 M pH 5 MES buffer. The activated Cy5 was then added to the Chit−IOCO−MTX mixture and allowed to stir overnight. The reacted Chit−IOCO−MTX−Cy5 and Chit−IOCO−Cy5 solutions were centrifuged, and the pellets were sonicated at an amplitude of 30 % for 2 min instead of the 60 % amplitude for 4.5 min used in the dispersion of Chit−IOCO. This was due to the findings that increasing power of sonication resulted in a significant loss of Cy5 fluorescence signals (Fig. S2). Thus, lower power and shorter sonication time were applied without compromising the size and homogenous dispersion of NPs.

The Chit−IOCO−Cy5 was also prepared as above but without addition of MTX. This protocol resulted in 67 wt % EE of MTX and 20.9 wt % EE of Cy5. Cy5 was conjugated to the chitosan forming Chit−IOCO−Cy5. Chit−IOCO−Cy5 with the size distribution shown in Fig. 1D was 162.97 nm in hydrodynamic size with a PDI of 0.16 and a positive surface charge of 9.8 mV. The cerium and iron concentration in Chit-IOCO−Cy5 were determined to be 3.85 % and 8 mass % respectively. Chit−IOCO−MTX−Cy5 (Fig. 1E) showed irregular shape with a ∼90 nm length under TEM (Fig. 1F) and the size distribution was shown in Fig. 1Gi. The hydrodynamic size of Chit−IOCO−MTX−Cy5 was 179.6 nm, with a low PDI of 0.06 and zeta potential of 9.17 mV (Fig. 1Gii). ICP analyses revealed that Chit−IOCO−MTX consisted of 4.96 % cerium and 8.8 % iron (Fig. 1Giii). Chit−IOCO−MTX−Cy5 with different iron (Fe) concentrations were prepared on MRI phantoms as illustrated in Fig. 1H for the evaluation of MR imaging efficacy. The signal intensity of T_2_-weighted MR scans decreased with increasing Fe concentration (Fig. 1I). T_2_ from each phantom was automatically generated by the operating system and was used to plot 1/T_2_ against Fe concentration. The transverse relaxivity (*r*_*2*_) defined as the slope from the linear regression, was determined as 409.5 mM^−1^ s^−1^ at 9.4 T (Fig. 1J).

### 3.2 *In vitro* studies

#### 3.2.1 Cytotoxicity effects of Chit−IOCO−MTX-Cy5

**Fig. 2:**
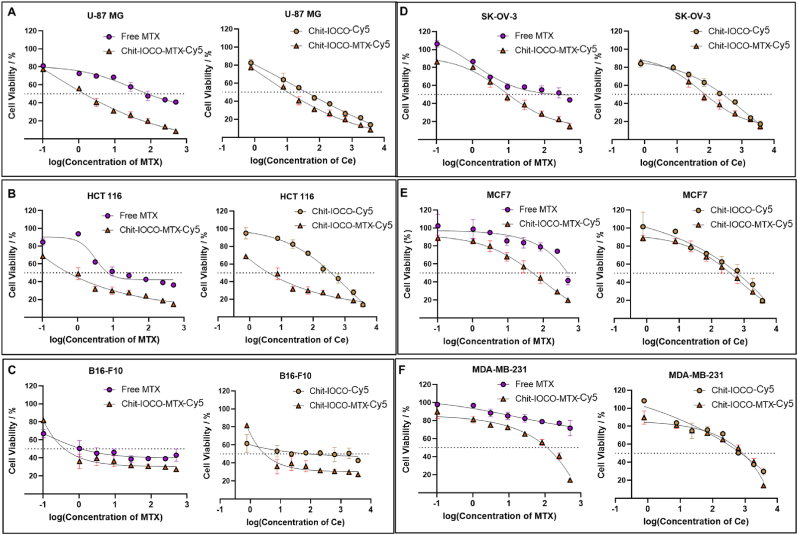
Anti-cancer effects of free methotrexate (MTX), Chit-IOCO-Cy5 and Chit-IOCO-MTX-Cy5 nanoparticles on different cancer cell lines. Cancer cells were treated with different concentrations of free MTX, Chit-IOCO-Cy5 and Chit-IOCO-MTX-Cy5 for 48 h. The fluorescence of non-treated cells was determined as 100 % cell viability. Sigmoidal dose-response curves of (A) U-87 MG, (B) HCT 116, (C) B16-F10, (D) SK-OV-3, (E) MCF7 and (F) MDA-MB-231 cells plotted against log concentrations of MTX and CO were produced from non-linear regression analyses using GraphPad Prism.

Six cancer cell lines were screened against free MTX, Chit−IOCO and Chit−IOCO−MTX to determine cell lines that were most responsive to the combination treatment of MTX and nanoceria. Viability of cells treated with Chit−IOCO−MTX were compared to those treated with equivalent doses of free MTX or Chit−IOCO to evaluate the synergistic anti-cancer activities of MTX and nanoceria. As shown in Fig. 2, glioma U-87 MG and colon cancer HCT 116 cells survival was less than 50 % at low concentrations of Chit−IOCO−MTX, and cell viability decreased with increasing concentrations (Fig. 2A and B). Melanoma B16-F10 cells demonstrated less than 50 % cell survival at low concentration, but the plot plateau with increasing dosage (Fig. 2C). Ovarian cancer SK-OV-3 cells showed dose-dependent cell survival but were less responsive than U-87 MG and HCT 116 cells evident by the higher concentration required to achieve 50 % cytotoxic effects (Fig. 2D). Breast cancer cells MCF7 and MDA-MB-231 were less responsive to nanoceria and MTX treatment, in particular MDA-MB-231 cells with >70 % viability even at the highest concentration of MTX at 227 μg/mL (Fig. 2E and F).

The synergistic effects of the MTX and nanoceria combination were assessed through the calculation of combination index (CI) determined from IC_50_ values (Table S1). The IC_50_ values of Chit−IOCO−MTX in U-87 MG cells were 2.22 μM (1.01 μg/mL) for MTX and 16.52 μM (2.31 μg/mL) for Ce. In HCT 116 cells, the IC_50_ for MTX and Ce were 2.49 μM (1.13 μg/mL) and 12.25 μM (1.72 μg/mL) respectively. The CI of Chit−IOCO−MTX was 0.247 in U-87 MG and 0.497 in HCT 116 cells.

#### 3.2.2 ROS modulating and apoptotic effects of Chit-IOCO-MTX-Cy5

**Fig. 3:**
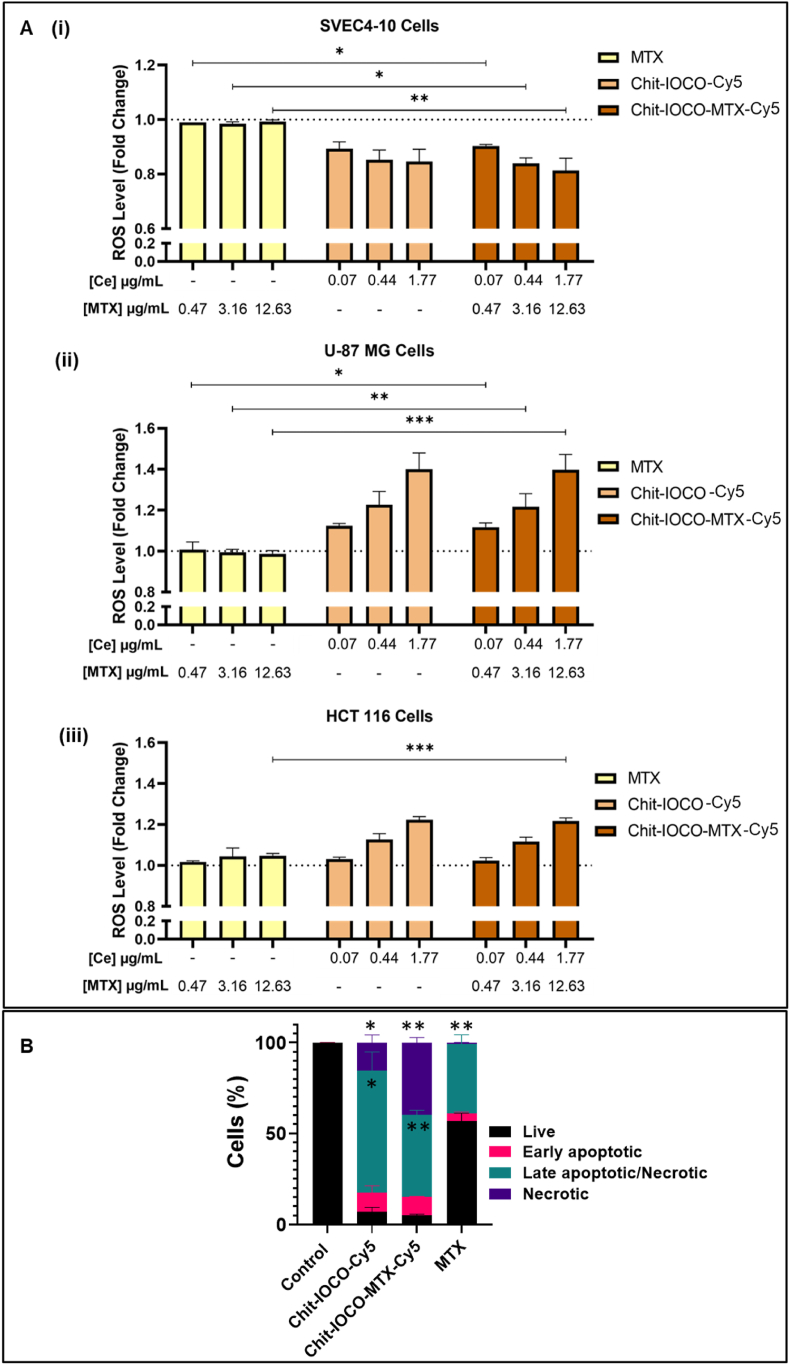
DCFDA assays to determine changes in intracellular ROS. (A) ROS of cells (SVEC4-10, U-87MG and HCT 116) treated with free methotrexate (MTX), Chit−IOCO or Chit−IOCO−MTX at different concentrations for 8 h. Fold change of intracellular ROS level was defined as fluorescence intensities of treated wells normalised against control wells. ^#^p ≤ 0.05, ^##^p ≤ 0.01, ^####^p ≤ 0.0001, Student's t-test. ∗p ≤ 0.05, ∗∗p ≤ 0.01, ∗∗∗p ≤ 0.001, Student's t-test comparing Chit−IOCO−MTX to MTX and to Chit−IOCO. Apoptotic effects of NPs. (B) Flow cytometry analysis of U87MG cells treated with NPs to determine stages of apoptosis. ∗ Compared to the respective stages of apoptosis cell population of the control group: ∗p < 0.05, ∗∗p < 0.01.

U-87 MG and HCT 116 cells demonstrating excellent anti-cancer responses to Chit−IOCO−MTX along with non-malignant SVEC4-10 cells were tested for intracellular ROS levels to investigate the role of nanoceria in modulating ROS responding to cellular environments. The intracellular ROS DCFDA assay parameters were first optimised as shown in Fig. S3A. Cells incubated with DCFDA reagent in DPBS supplemented with 10 % FBS demonstrated significantly higher fluorescence intensities in comparison to DCFDA in complete DMEM with phenol red. Higher fluorescence signals were also obtained with 45 min incubation time instead of 30 min. Therefore, we decided to perform ROS assay with DCFDA in DPBS and DCFDA incubation time of 45 min. It was found that the basal ROS levels in proliferating cells, in the absence of any trigger or treatments, were significantly higher in cancer cells than in endothelial cells (Fig. S3B). Upon administration of treatment, no changes in ROS levels were observed in MTX treated groups in all three cell lines (Fig. 3A). Notably, 0.07, 0.44 and 1.77 μg/mL nanoceria in Chit−IOCO and Chit−IOCO−MTX scavenged ROS in SVEC4-10 cells. In contrast, the identical amount of nanoceria forced ROS accumulation in U-87 MG cells. For HCT 116 cells, significant increased production of ROS was detected at 1.77 μg/mL nanoceria. Hence, considering the excellent cytotoxicity and greater ROS generating effects upon treatment with Chit−IOCO−MTX, U-87 MG cell line was selected for tumour development in further *in vitro* and *in vivo* studies. U-87MG cells were incubated with Chit-IOCO-Cy5, Chit−IOCO−MTX-Cy5 and MTX whereby apoptotic and necrotic cell populations were evaluated using the Annexin V-FITC Apoptosis Detection Kit. The untreated cells (control) displayed a viable cell population of 99.7 % whilst the apoptotic and necrotic cells were insignificant (Fig. 3B). Post NPs treatment, live cell population decreased significantly to <10 % for both Chit-IOCO-Cy5 and Chit-IOCO-MTX-Cy5. Contrastingly, cells treated with MTX displayed a cell viability of 56.7 %, clearly indicating the enhanced cytotoxicity of NPs in comparison to MTX only. Additionally, cells treated with Chit-IOCO-Cy5 and Chit-IOCO-MTX-Cy5 were mostly in the late apoptotic/necrotic stage at 66.7 % and 45.2 % respectively. U-87 MG cells treated with Chit-IOCO-MTX-Cy5 had the highest cell population of necrotic cells at 39.8 % in comparison to the other treated groups (MTX: 0.8 %; Chit-IOCO: 15.6 %) suggesting the potent cytotoxicity of NPs.

#### 3.2.3 MTX as active targeting ligand & biocompatibility of nanoparticles

**Fig. 4:**
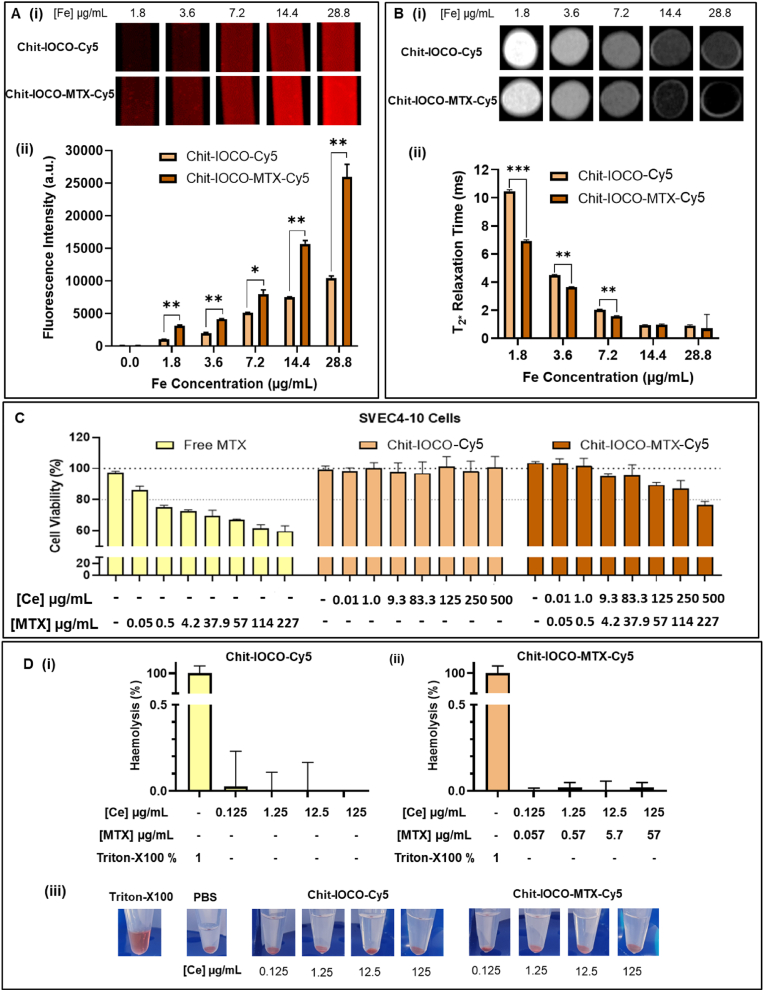
Cellular uptake of nanoparticles with and without targeting ligand methotrexate (MTX). U-87 MG cells were treated with different iron (Fe) concentrations of Chit−IOCO−Cy5 and Chit−IOCO−MTX−Cy5 for 8 h at 37 °C with 5 % CO_2_, harvested for fluorescence and magnetic resonance (MR) imaging. (A) Cellular uptake measured by Cy5 fluorescence intensities. (i) Fluorescence images of harvested cells scanned on a Sapphire Biomolecular Imager with a 658 nm excitation laser coupled to a 670–750 nm filter. (ii) Fluorescence intensities of harvested cells determined from taking region of interest (ROI) measurement on ImageJ. (B) Cellular uptake measured by MR imaging. (i) T_2∗_-weighted MR scan images of prepared phantoms. (ii) Relaxation time (T_2∗_) that were automatically generated from T_2∗_-weighted scans by the operating system. ∗p ≤ 0.05, ∗∗p ≤ 0.01, Student's t-test comparing Chit−IOCO−MTX to Chit−IOCO. *In vitro* biocompatibility of Chit−IOCO and Chit−IOCO−MTX nanoparticles. (C) Cytotoxicity of methotrexate (MTX), Chit−IOCO and Chit−IOCO−MTX in SVEC4-10 cells. Cells were incubated with different concentrations of nanoparticles treatment for 48 h. The fluorescence of non-treated cells was determined as 100 % cell viability. (D) Haemolysis assay of red blood cells incubated with increasing concentrations of Chit−IOCO−Cy5 (i) and Chit−IOCO−MTX−Cy5 (ii) for 1 h at 37 °C. iii) Representative images. (For interpretation of the references to colour in this figure legend, the reader is referred to the Web version of this article.)

The performance of MTX ligand targeting folate transporters in U-87 MG cells were assessed by comparing cellular uptake of Chit−IOCO−Cy5 and Chit−IOCO−MTX−Cy5. Cells treated with NPs were harvested and prepared in MRI phantoms as illustrated in Fig. 4A. Brighter fluorescence signals were observed in cells treated with MTX-, Cy5-conjugated NPs (Fig. 4Ai) with significantly higher fluorescence intensities measured in all concentrations of Chit−IOCO−MTX−Cy5 in comparison to Chit−IOCO−Cy5 (Fig. 4Aii). Visibly darker signals were observed in Chit−IOCO−MTX−Cy5 treated cells in T_2∗_- weighted MRI scans at all concentrations (Fig. 4Bi). Significantly shorter T_2∗_ relaxation time was determined in MTX conjugated NPs at 1.8, 3.6 and 7.2 μg/mL Fe (Fig. 4Bii).

The *in vitro* biocompatibility of Chit−IOCO and Chit−IOCO−MTX were assessed in non-disease SVEC4-10 cells and human red blood cells (RBCs) prior to commencement of animal studies. Bare MTX was cytotoxic to SVEC4-10 cells with cell viability less than 80 % and further decreasing from 0.5 μg/mL. In comparison, Chit−IOCO demonstrated excellent cytocompatibility even at high concentrations up to 500 μg/mL nanoceria. Chit−IOCO−MTX revealed improved biocompatibility at all MTX concentrations up to 114 μg/mL, with less than 80 % viability at the highest concentration tested (Fig. 4C). Haemolysis assay was performed on human RBCs treated with Chit−IOCO and Chit−IOCO−MTX. All NPs treated groups had less than 0.3 % haemolysis, indicating that the Chit−IOCO and Chit−IOCO−MTX have excellent haemocompatibility (Fig. 4D).

### 3.3 *In vivo* studies

#### 3.3.1 Biosafety assessment

**Fig. 5:**
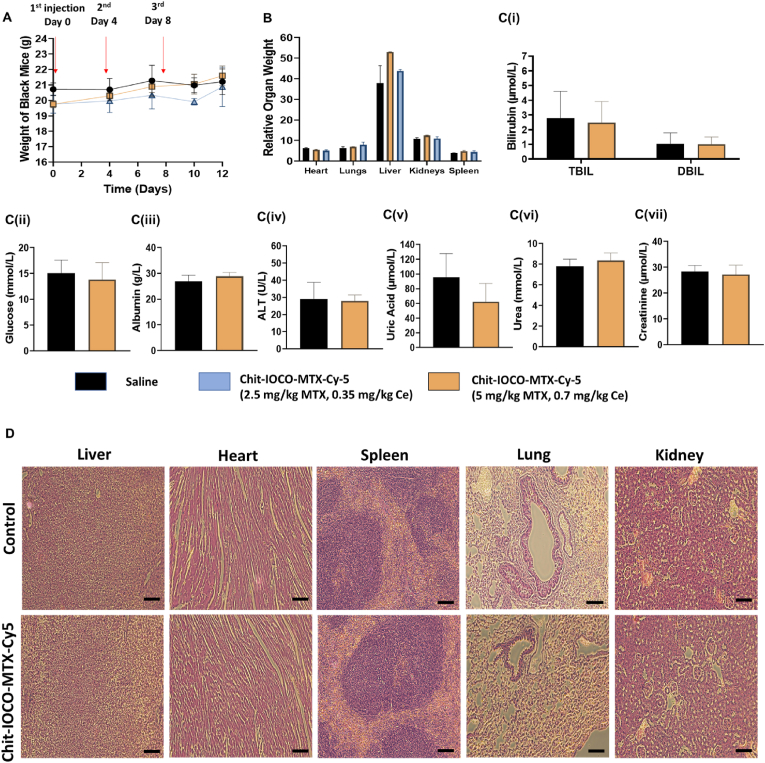
*In vivo* biosafety studies of Chit−IOCO−MTX−Cy5 in female C57BL/6J mice. Mice were injected intravenously via tail vein with 0.9 % saline (control group), 2.5 and 5 mg/kg MTX equivalent (0.35 and 0.7 mg/kg equivalent Ce) of Chit−IOCO−MTX−Cy5 at Day 0, 4 and 8. (A) Average body weight of mice monitored twice a week. (B) Relative organ weights (organ weight to body weight ratios) were determined as mg organ weight/g terminal body weight of mice. (C) Biochemical analysis of serum from C57BL/6J mice. Blood samples were collected at Day 12 via cardiac puncture. Purified serum samples from whole blood collected were tested for (i) Bilirubin, (ii) Glucose, (iii) Albumin, (iv) Alanine transaminase (ALT), (v) Uric acid. (vi) Urea and (vii) Creatinine. (D) Histological images of tissues stained with H & E indicating the status of major organs after animals were sacrificed at Day 12 post i.v. injection. Scale bar: 100 μm.

The biosafety of Chit−IOCO−MTX−Cy5 was assessed by monitoring the wellbeing of non-disease C57BL/6J mice intravenously injected with NPs. No drastic changes in the mice body weight were observed (Fig. 5A). Fig. 5B showed the terminal relative weights of organs including heart, lungs, liver, kidneys and spleen. The organ weight to body weight ratios of heart, lungs, kidneys and spleen were at similar levels. However, liver was a lot heavier from mouse treated with 5 mg/kg MTX equivalent of Chit−IOCO−MTX−Cy5. Subsequently, biochemical analysis of liver function markers such as bilirubin, alanine transaminase (ALT), glucose and albumin and kidney function markers including uric acid, urea and creatinine were measured accordingly (Fig. 5Ci-vii). In the NP treatment groups, total bilirubin count (TBIL) decreased slightly and direct bilirubin count (DBIL) was similar in range in comparison to controls. A slight decrease in glucose, ALT and creatinine was observed in treatment groups. A small increase in albumin and urea was observed in NP treatment group. These slight changes however were not significant compared to the healthy control. Whilst a decrease in uric acid was observed in correlation to controls, uric acid levels were still within acceptable range. Tissues of main organs were stained with H & E and imaged. Fig. 5D display liver, heart, spleen, lung, and kidney of Chit-IOCO-MTX-Cy5 treated and control mice. No significant anomaly or inflammation was observed at repeated doses of NPs.

#### 3.3.2 Tumour growth inhibition

**Fig. 6:**
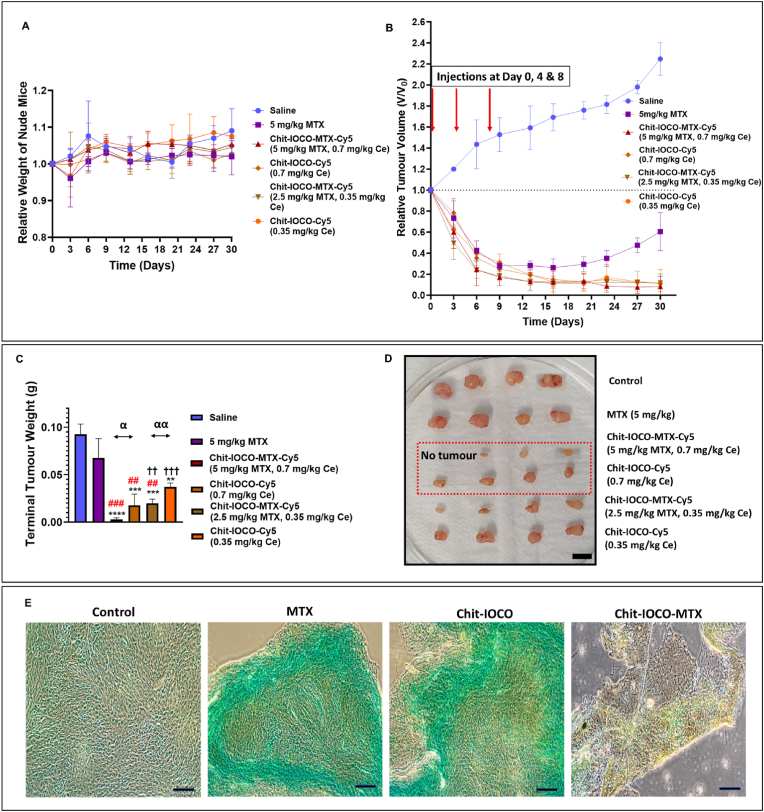
*In vivo* therapeutic effects of free MTX, Chit-IOCO-Cy5 and Chit-IOCO-MTX-Cy5 nanoparticles in U-87 MG tumour-bearing BALB/C mice. Mice were injected intravenously with 0.9 % saline (control group), 5 mg/kg bare methotrexate (MTX), 2.5 and 5 mg/kg MTX equivalent (0.35 and 0.7 mg/kg equivalent Ce) of Chit-IOCO-MTX-Cy5 and 0.35 and 0.7 mg/kg equivalent Ce of Chit-IOCO-Cy5 at Day 0, 4 and 8. (A) Average body weight of mice monitored twice a week during the treatment. Mice body weight analysed with repeated measures one-way ANOVA with Tukey's multiple comparisons demonstrated no significant changes in all treatment groups across the experimental period. (B) Changes in tumour volume during the experiment period. Relative tumour volume was defined as tumour volume (V_0_) measured normalised to tumour volume at Day 0 before administration of treatment (V_0_). (C) Terminal tumour weight and (D) representative images of tumour from each group. Scale bar: 10 mm. One-way ANOVA with Tukey's Multiple Comparisons Test. Compared to Saline: ∗∗p < 0.01, ∗∗∗p < 0.001, ∗∗∗∗p < 0.0001. Compared to MTX: ##p < 0.01, ###p < 0.001. Compared to Chit-IOCO-MTX-Cy5 (5 mg/kg MTX): ††p < 0.01, †††p < 0.001. Student's t-test comparing Chit−IOCO−MTX-Cy5 to Chit−IOCO-Cy5. α < 0.05, αα < 0.01. (E) Histological images of tumour tissues stained with TUNEL. Scale bar: 100 μm.

U-87 MG cells were inoculated subcutaneously into the right flank of BALB/C mice and treatment administration commenced when tumour volume reached >100 mm^3^. Tumour-bearing mice were intravenously injected with saline as control and free MTX, Chit−IOCO−Cy5 and Chit−IOCO−MTX−Cy5 to compare the cancer-killing effects of Chit−IOCO−MTX−Cy5 to bare MTX and Chit−IOCO−Cy5. Repeated measures one-way ANOVA with Tukey's multiple comparisons revealed no drastic changes in the mice body weight were observed on the tumour-bearing mice (Fig. 6A). As shown in Fig. 6B and C, in contrast to saline group, all treated groups demonstrated significant decrease in tumour volume across treatment period except MTX treated group. After the final injection, tumours in MTX treated group re-grow and increased continually over time in contrast to the 3 NP treatment groups. Whilst a higher concentration of Chit-IOCO-Cy5 (0.7 mg/kg of Ce) group revealed an enhanced reduction in tumour volume, Chit−IOCO−MTX−Cy5 (5 mg/kg MTX) group displayed augmented cytotoxicity whereby one mouse had no visible tumour at the end point of experiment which suggested complete eradication of tumour. Chit−IOCO−MTX−Cy5 (2.5 mg/kg MTX) displayed a significant difference in terminal tumour weight in comparison to nanoceria without MTX conjugation. Both concentrations of Chit-IOCO-MTX-Cy5 groups displayed a reduced terminal tumour weights in comparison to the corresponding Chit-IOCO-Cy5 group, highlighting the improved therapeutic efficacy with MTX conjugation. Fig. 6D demonstrates representative tumours treated with saline, MTX (5 mg/kg MTX), Chit-IOCO-Cy5 (0.7 mg/kg Ce) and Chit-IOCO-MTX-Cy5 NPs (5 mg/kg MTX) which are stained with TUNEL. Saline treated group depicted a healthy tumour cell morphology. MTX and Chit-IOCO-Cy5 treated groups depicted a shrunken tumour cell morphology accompanied with shortened chromatin and some apparent brown stains. Chit-IOCO-MTX-Cy5 group revealed substantial damages to the tumour represented by cell shrinkage and fragmentation with numerous distinct brown stains.

#### 3.3.3 Targeting and imaging efficacy

**Fig. 7:**
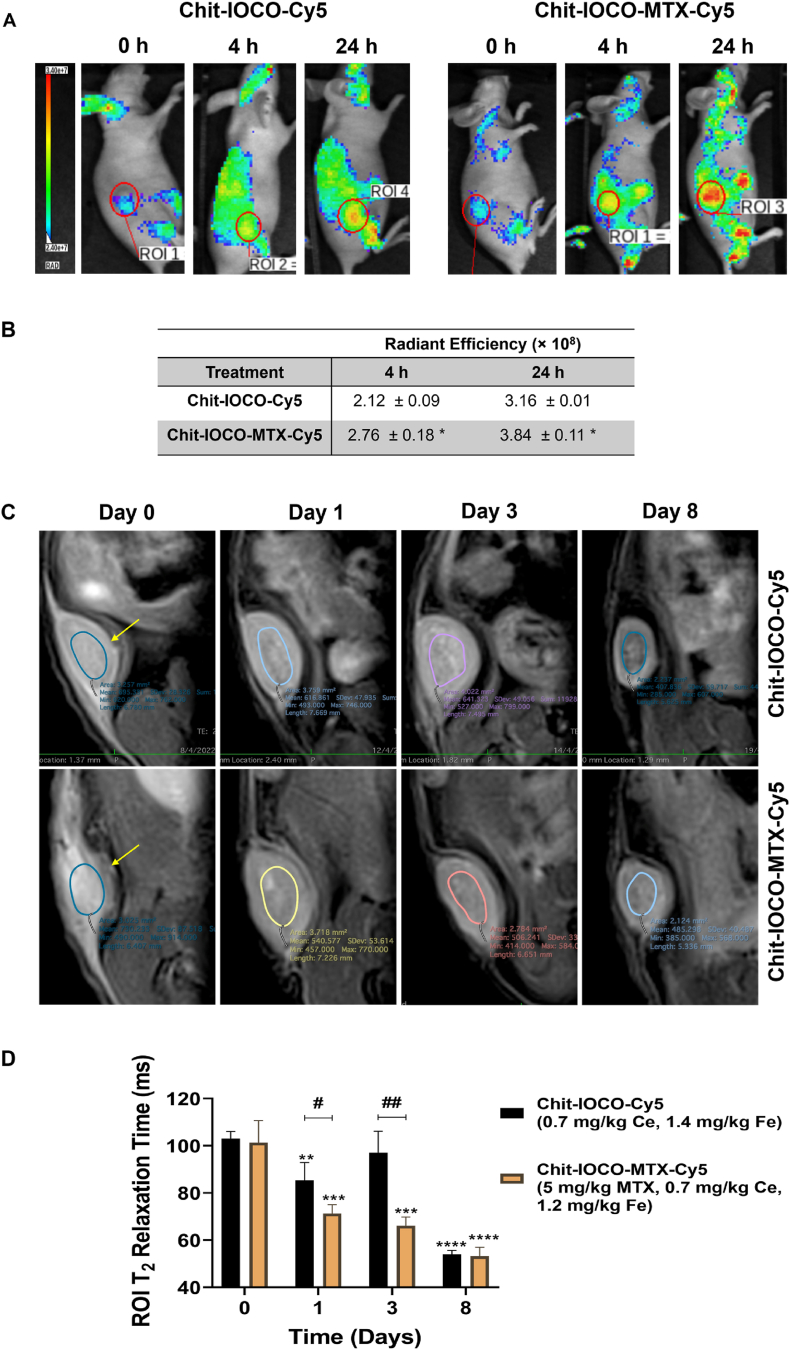
*In vivo* tumour targeting efficacy of Chit-IOCO−Cy5 and Chit-IOCO−MTX−Cy5 nanoparticles in U-87 MG tumour-bearing BALB/C mice. Mice were injected intravenously (IV) via tail vein with Chit-IOCO−Cy5 and Chit-IOCO−MTX−Cy5 at Day 0, 4 and 8. Fluorescence images of mice taken using IVIS Lumina X5 at 4 and 24 h post iv injections and processed with Aura Imaging Software. (A) Representative images were shown with white circles as regions of interest (ROI) where tumours were located. (B) The ROI values were calculated and presented as the total radiant efficiency. ∗p < 0.05, Student's t-test comparing Chit−IOCO−MTX to Chit−IOCO. Mouse tumours were imaged using a 7 T MRI before (Day 0), 1- (Day 1), 3-day (Day 3) after first injection and 5-day (Day 8) after second intravenous injection of Chit-IOCO−Cy5 (1.4 mg/kg Fe) and Chit-IOCO−MTX−Cy5 (1.2 mg/kg Fe). (C) Representative MR images of mice. Coloured circles represented ROI taken for T2 determination. Yellow arrows denote tumour. (D) Transverse relaxation time T_2_ values were automatically generated by the operating system using region of interest (ROI) function on the tumours. ∗∗p ≤ 0.01, ∗∗∗p ≤ 0.001, ∗∗∗∗p ≤ 0.0001, one-way ANOVA with Dunnett's multiple comparisons comparing T_2_ values of Day 1, 3 and 8 to Day 0. #p ≤ 0.05, ##p ≤ 0.01, Student's t-test comparing Chit−IOCO−MTX−Cy5 to Chit−IOCO−Cy5. (For interpretation of the references to colour in this figure legend, the reader is referred to the Web version of this article.)

The U-87 MG tumour-bearing BALB/C mice were imaged under IVIS and MRI to determine the targeting and imaging performances of Chit−IOCO−MTX. As shown in Fig. 7, significantly higher fluorescence signals were observed at the tumour region of interest (ROI) of Chit−IOCO−MTX−Cy5 treated mouse in relative to Chit−IOCO−Cy5 at 4 and 24 h after IV administration of NPs.

The tumour-bearing mice were imaged using a 7 T MRI before (Day 0), 1- (Day 1), 3-day (Day 3) after first injection and 5-day (Day 8) after second injection of Chit-IOCO−Cy5 and Chit-IOCO−MTX−Cy5. The representative T_2_-weighted MR images and T_2_ values of tumours ROI were shown in Fig. 7C and D. T_2_ was significantly shortened in all mice treated with Chit-IOCO−MTX−Cy5 at all timepoint. 1-Day after first injection, Chit−IOCO−MTX−Cy5 demonstrated significantly higher NPs accumulation at tumour sites as reflected in the significantly shortened T_2_ in comparison to Chit−IOCO−Cy5. 3 days after first administration of NPs, the T_2_ of Chit−IOCO−Cy5 became longer and not significantly different from the T_2_ at Day 0. At Day 8 of the study, which was also 5 days after second injection, excellent accumulation of NPs both with and without MTX was observed in all mice. The changes in tumours across time could also be visualised on the MR images. Tumours with Chit−IOCO−MTX−Cy5 treatment decreased in size at Day 3 and 8 whilst the shrinking of tumours injected with Chit−IOCO−Cy5 could only be identified at Day 8 imaging.

## 4 Discussion

### 4.1 Synthesis and characterisation of NPs

In this study, the chitosan nanocomposites containing iron oxide and cerium oxide NPs were successfully synthesised. Chitosan, a natural polysaccharide, has been extensively investigated for biomedical and biopharmaceutical applications, including the preparation of micro- and nano-particles as anticancer drug carrier [61]. Employing chitosan promotes the sourcing of cost-effective and environmentally friendly materials as chitin is the second most abundant natural occurring polymer after cellulose [62,63]. Chitosan as a biocompatible natural polymer deemed GRAS (Generally Recognised as Safe) by FDA for specific applications, demonstrates excellent biodegradability and chemical versatility with low or non-toxicity indicating that it is a promising and valuable nanocarrier. Garg and co-workers demonstrated that chitosan-based NPs are effective agents in drug delivery and targeting, showing potential in various drug delivery systems including ocular, per-oral, pulmonary, nasal, mucosal, gene, buccal, vaginal, vaccine delivery and cancer therapy [64].

The conjugation of Cy5 and/or MTX to Chit−IOCO was optimised in terms of mole ratio of reactants and reacting conditions. The increase in NPs size (from 158.1 to 162.97 nm) and decrease in surface charge (from 29.2 to 9.8 mV) demonstrated successful loading of Cy5 to Chit−IOCO. Chit−IOCO−MTX−Cy5 with a size of 179.6 nm and slight decrease in surface charge (9.17 mV) indicated successful conjugation of MTX. The decrease in surface charge was due to the formation of neutral amide bonds between the positively charged chitosan's amino groups and the negatively charged carboxylic groups of both MTX and Cy5 [65]. The loading of Cy5 and MTX potentially had affected the encapsulation of IO−PAA and CO−TSC in chitosan as reflected in the mass percentage, ∼8 % Fe and ∼4 % Ce that were both lower than Chit−IOCO (13 % loading for both Fe and Ce [42]). IO−PAA and CO−TSC were loaded onto chitosan via electrostatic attractions between the oppositely charged groups. The positive charge of chitosan was gradually decreased in subsequent synthesis steps leading to potential loss of anionic IO−PAA and CO−TSC from chitosan. The sizes of Chit−IOCO−Cy5 and Chit−IOCO−MTX−Cy5 that were within the range of 10–500 nm could contribute to passive drug targeting through EPR activity [66]. Enhanced cellular uptake was also anticipated given the fact that chitosan-based nanomaterials are positively charged in slightly acidic solution [65]. It was reported that tumour cells specific uptake was achieved with NPs being positively-charged in acidic TME whilst becoming neutral and sparing normal cells under normal physiological conditions [67]. Hence, the nanoscale sized Chit−IOCO−MTX−Cy5 with positive surface charge under acidic environment presented itself favourable for cellular uptake.

Chit-IOCO−MTX−Cy5 with increasing concentrations of Fe demonstrated decreasing intensities in T_2_-weighted image. This was anticipated from iron oxide being a negative contrast agent that shorten the transverse relaxation time T_2_ of surrounding protons [68]. *r*_*2*_ of Chit−IOCO−MTX-Cy5 was calculated to be 409.5 mM^−1^ s^−1^ at 9.4 T, which was interestingly, an improvement from previously reported 308 mM^−1^ s^−1^ despite the increase in NPs size [42]. This could be resulted from IO−PAA not fully encapsulated within the nanocomposites and as the positive charge of chitosan decreased, increased number of IO−PAA particles were presented on the surface of NPs. With more iron oxide on the surface, higher efficiency in the transferring of magnetic moments to nearby protons was achieved in the presence of external magnetic field. The transverse relaxivity of Chit−IOCO−MTX−Cy5 was also superior to clinically approved iron oxide contrast agents such as Feridex® (*r*_*2*_ = 307.5 mM^−1^ s^−1^ at 9.4 T [69], *r*_*2*_ = 93 mM^−1^ s^−1^ at 3 T [70]), Resovist® (*r*_*2*_ = 374.6 mM^−1^ s^−1^ at 9.4 T [71], *r*_*2*_ = 143 mM^−1^ s^−1^ at 3 T [70]), FeREX™ (*r*_*2*_ = 283.6 mM^−1^ s^−1^ at 9.4 T [69], *r*_*2*_ = 160.1 mM^−1^ s^−1^ at 3 T [72]) and Feraheme® (*r*_*2*_ = 89 mM^−1^ s^−1^ at 3 T [73]). Thus, Chit−IOCO−MTX−Cy5 demonstrated excellent MRI contrast capability that was comparable to commercially available products.

### 4.2 *In vitro* study

#### 4.2.1 Chit−IOCO−MTX-Cy5 as cancer therapeutic agent

Preliminary screening of a panel of cancer cells against free MTX, Chit−IOCO and Chit−IOCO−MTX revealed variations in cellular response to therapeutic agents. All six cell lines tested were previously either shown to have overexpressed levels of FR-α [74,75] or responsive to treatments targeting folate transporters [76,77]. However, breast cancer cell line MDA-MB-231 was not responsive to MTX treatment with no differences in the cell survival when treated with NPs with or without MTX conjugation. This was consistent with previous studies demonstrating MDA-MB-231 as methotrexate resistant cell line [78,79]. MDA-MB-231 cells were lack of RFC expression which is one of the major transmembrane transporters of MTX [80,81]. Moreover, some studies have found that nanoparticles targeting folate receptors demonstrate lower uptake in MDA-MB-231 and MCF-7 cells [82]. This reduced uptake is likely due to the lower expression of folate receptors in MDA-MB-231 and MCF-7 compared to other breast cancer cell types, leading to decreased internalization of the nanoparticles. This observation supports the lack of significant difference in cell viability between the targeted and non-targeted groups. In contrast, a recent study by Unida et al. successfully showed that folate-functionalised DNA nanocages loaded with Dox effectively killed MDA-MB-231 cells compared to their non-targeted counterparts [83]. This difference in cytotoxicity highlights that the physicochemical properties of the nanoparticles, including size, surface charge, and coating, play a significant role in their interaction and uptake by cells. Interestingly, the cell viability of MCF-7 decreased to <50 % only at the highest tested concentration of MTX despite being reported as MTX-responsive cells [84],

Melanoma B16-F10 cells were effectively killed at low concentrations of treatments but the anti-cancer effects were not dose responsive that the dose-response curve plateau with increasing concentrations of both MTX and nanoceria. This indicated that the maximal therapeutic effect had been achieved hence additional dose would not improve treatment outcome [85].

The three remaining cell lines including glioma cells U-87 MG, colon cancer cells HCT 116 and ovarian cancer cells SK-OV-3 all demonstrated increased cell death when treated with Chit−IOCO−MTX-Cy5 in comparison to free MTX and Chit-IOCO-Cy5 without MTX conjugation. The calculated combination index of less than 1 inferred that MTX and nanoceria acted synergistically [86] in U-87 MG and HCT 116 cells with a ∼4× and ∼2× dose shift respectively. This data suggests that the conjugation of NPs to MTX was essential to result in an enhanced cytotoxic effect in cancer cells. This indicated that incorporation of MTX ligand which is a folic acid analogue efficiently targeted cancer cells with overexpressed levels of FR via FR mediated endocytic and intracellular folate dependent metabolic pathways resulting in augmented cytotoxicity in cancer cells. Similarly, Chit-IOCO-Cy5 displayed dose-dependent killing of U-87MG cells as the cerium oxide concentration increased suggesting the anticancer efficacy of nanoceria. Previously reported studies have demonstrated that the oxidation states of nanoceria is highly influenced by the pH levels, whereby a more acidic tumour environment results in higher ROS generation and oppositely for ROS scavenging [24,87]. Thus, the difference in acid-base environment between normal and tumour tissues can be exploited in anticancer treatment strategy via oxidative stress resulting in apoptosis of cancer cells while protecting normal tissues via attenuation of free radicals. Hence, U-87 MG and HCT 116 cells that revealed synergistic anti-cancer effects of nanoceria/MTX were chosen for subsequent studies.

#### 4.2.2 ROS modulating & apoptotic effects of Chit-IOCO-MTX-Cy5

The approach of establishing nanoceria-based NPs as cancer therapeutic agent is based on the ROS modulating activities of nanoceria. The cancer cells U-87 MG and HCT 116 had significantly higher ROS signals than the endothelial cells in the absence of treatments or triggers. Cancer cells typically have higher basal ROS levels to support tumour growth and higher antioxidant activities to maintain ROS levels below cytotoxic threshold, making the cancer cells susceptible to therapeutic agent that results in ROS accumulation [10,88]. In endothelial cells where the basal ROS level was under normal redox homeostasis without oxidative stress, Chit−IOCO and Chit−IOCO−MTX slightly decreased the levels of intracellular ROS down to ∼0.8 fold of non-treated cells. Cancer cells on the other hand, exhibits aberrant redox balance [10,88] with slightly acidic pH due to the excess acidic metabolic waste from uncontrolled cell proliferation [89,90]. Under these environments, Chit−IOCO and Chit−IOCO−MTX induced a 1.4-fold and 1.2-fold increase of ROS in U-87 MG and HCT 116 cells respectively. This forced ROS accumulation induced oxidative stress that triggered cancer cell apoptosis which resulted in a high population of U-87MG cells in the late apoptotic and necrotic stage [11,12]. The intracellular ROS studies revealed that the MTX dosage tested did not interfere with redox balance and this was further corroborated by the findings that NPs with or without MTX conjugation demonstrated similar changes of ROS levels in both cancer and non-malignant cells. However, the activities of MTX in mediating ROS could be dependent on cell lines and dosage. AlBasher and co-workers reported that MTX contributed to oxidative stress provoking apoptosis in SK-OV-3 cells [91]. Thus, the activities of MTX in redox environment should be evaluated under varying circumstances. In this study, ROS accumulation in cancer cells arise from the increase in Chit-IOCO concentrations.

As U-87 MG cell demonstrated stronger synergistic dose shift effect and significantly higher ROS production at lower concentrations of nanoceria, it was concluded that the developed Chi-IOCO-MTX-Cy5 was most effective on U87 MG-based tumor or glioblastoma. Therefore, U87 MG was finalised as the cell line used to develop tumour models for *in vivo* experiments.

Additionally, the observed differences in apoptotic cell populations among MTX, Chit-IOCO, and Chit-IOCO-MTX carry significant implications for therapeutic strategies. The enhanced induction of both apoptosis and necrosis by Chit-IOCO-MTX suggests a more effective mechanism for targeting and eliminating cancer cells. This heightened apoptotic response, particularly with a substantial percentage of cells entering late apoptosis, indicates that Chit-IOCO-MTX not only facilitates cell death but may also prevent the development of resistance often seen with conventional therapies. Furthermore, the increased necrotic cell population associated with Chit-IOCO-MTX may disrupt the tumour microenvironment, potentially limiting cancer cell survival and proliferation. Such dual mechanism of action—promoting both apoptosis and necrosis—could enhance overall tumour eradication, making Chit-IOCO-MTX a promising candidate for improving clinical outcomes in cancer treatment. The ability to induce significant cell death while minimizing impacts on surrounding healthy tissue underscores the potential of this targeted approach to optimise therapeutic efficacy and reduce side effects associated with traditional chemotherapeutics.

#### 4.2.3 Targeting efficacy of MTX & biocompatibility

Chemotherapeutic agent MTX being a structural analogue of folate, is exploited as a ligand actively targeting folate transporters [40,92]. Enhanced uptake of Chit−IOCO−MTX−Cy5 in comparison to Chit−IOCO−Cy5 was detected in U-87 MG cells via fluorescence and MR imaging. This indicated that incorporation of MTX ligand efficiently targeted cells with overexpressed levels of folate transporters. The results also further corroborated the MR contrast efficacy of both Chit−IOCO−Cy5 and Chit−IOCO−MTX−Cy5 *in vitro*. Strong MRI T_2_∗ effects were observed whereby the T_2_∗ relaxation times were significantly shortened in U-87 MG cells treated with MTX conjugated NPs in contrast to non-targeted NPs. These results suggest effective targeting of the nanoparticles when using MTX.

Biocompatibility is a crucial factor in establishing NPs as therapeutic agents that the NPs should deliver targeted therapeutic effects with minimal adverse events [[93], [94], [95]]. Chit−IOCO demonstrated no cytotoxicity at all concentrations tested (up to 500 μg/mL nanoceria) in endothelial cells. MTX, known to have a narrow therapeutic window requiring near-lethal dose for effective cancer killing [[96], [97], [98]], was toxic to endothelial cells from 0.5 μg/mL MTX. When MTX was conjugated to Chit−IOCO, a drastic improvement in cytocompatibility was observed in comparison to free MTX drugs that cell viability was >80 % when exposed to MTX concentrations up to 114 μg/mL. These results suggested that the loading of MTX onto chitosan nanocarrier could potentially decrease the adverse effects associated when healthy tissues were exposed to bare MTX. This improvement in cytocompatibility could be resulted from the changes in MTX structure upon formation of amide bonds [99]. However, as synergistic anti-cancer effects were observed in U-87 MG and HCT 116 cells, the structural-activity relationship of MTX conjugated to NPs need to be further evaluated to ensure the structural modifications would achieve reduction in side effects whilst enhancing therapeutic efficacy [100]. Haemocompatibility of NPs is one of the most important criteria to be assessed prior to *in vivo* applications and can be evaluated by haemolysis assay [101]. No significant haemolysis was identified in Chit−IOCO−Cy5 and Chit−IOCO−MTX−Cy5 treated human RBCs. The excellent haemocompatibility of both Chit−IOCO−Cy5 and Chit−IOCO−MTX−Cy5 suggested that they were suitable for administration into systemic circulation.

### 4.3 *In vivo* study

#### 4.3.1 Biosafety assessment of Chit−IOCO−MTX−Cy5

The acute biosafety of Chit−IOCO−MTX−Cy5 was examined in non-disease mice exposed to a total of three administrations of NPs. One animal displayed signs of deteriorating health after the final injection of Chit−IOCO−MTX−Cy5 at a concentration of 5 mg/kg MTX, 0.7 mg/kg Ce and no apparent adverse events were identified with the remaining animals. The relative organ weight was used for the macroscopic evaluation of toxicities by measuring the organ weight with respect to total body weight of vital organs including heart, lungs, liver, kidneys and spleens and comparing the NPs treated animals to control animals [102]. Whilst mice injected with Chit−IOCO−MTX−Cy5 at 2.5 mg/kg MTX did not show drastic differences in organ weights, mice treated with Chit−IOCO−MTX−Cy5 at 5 mg/kg MTX, 0.7 mg/kg Ce revealed slightly heavier terminal liver weight. Biochemical analysis of liver and kidney function markers were analysed and overall, these levels were in the acceptable range which indicates that repeated infusion of Chit−IOCO−MTX−Cy5 at 5 mg/kg MTX did not result in hepatic or renal damage. Additionally, no distinct toxicity was observed in tissues of major organs.

#### 4.3.2 Anti-tumour efficacy of Chit−IOCO−MTX−Cy5 and Chit−IOCO−Cy5

The *in vivo* therapeutic efficacy of Chit−IOCO−MTX−Cy5 was compared to free MTX and Chit−IOCO−Cy5 by comparing change in tumour volume over time. The body weight of tumour-bearing mice was not drastically changed throughout the experiment period indicating the absence of acute adverse effects. In comparison to saline group where the tumour continued to grow, all treated groups except free MTX demonstrated significant reduction in tumour growth. Particularly Chit-IOCO-MTX at 5 mg/kg resulted in superior tumour growth inhibition suggesting that MTX and nanoceria synergistically enhanced anti-cancer abilities in comparison to free MTX and nanoceria without MTX conjugation. Interestingly, after the final injection, tumours in MTX treated group re-grow and increased continually over time in contrast to the 3 NP treatment groups. As Chit−IOCO without MTX conjugation demonstrated excellent tumour growth inhibition in comparison to free MTX, co-administration of Chit−IOCO with decreased dosage of MTX could also be explored as an alternative synergistic treatment approach. Additionally, as *in vivo* studies were conducted in a subcutaneous U87MG model, further assessment of therapeutic efficacy of NPs will be evaluated in an orthotopic glioma model. Nonetheless, data obtained here demonstrated a proof-of-concept that our NPs displayed outstanding anticancer abilities.

#### 4.3.3 Tumour-targeting and imaging efficacy of Chit−IOCO−MTX−Cy5

The cancer targeting activities of Chit−IOCO−MTX−Cy5 were evaluated with both fluorescence and MR imaging. Under IVIS imaging, significant increase in fluorescence signals was observed within tumour ROI at both the 4 and 24 h timepoint in Chit−IOCO−MTX−Cy5 treated mice. The higher ROI signals indicated that increasing number of NPs with MTX conjugation were targeted to the tumour region post tail vein injections. Beyond 24 h of NPs injection, fluorescent signals could no longer be distinguished from background artefacts.

With a 7 T MRI preclinical scan system, T_2_ enhancement effects were observed at the tumours 24 h post-first injection of NPs with and without targeting ligand MTX suggesting that the NPs were both selectively and passively taken up by tumour cells. The T_1_-weighted images (data not shown) revealed that the NPs did not interfere with longitudinal relaxation of protons as the signal intensities and T_1_ relaxation time were not affected by administration of NPs. Significant difference in the shortening of T_2_ between Chit−IOCO−MTX−Cy5 and Chit−IOCO−Cy5 at 24 h indicated enhanced cellular uptake in the presence of MTX. Hence, the heightened anti-cancer abilities could be correlated to the enhanced retention of Chit-IOCO-MTX-Cy5 NPs in comparison to Chit-IOCO-Cy5. 72 h after first administration of NPs, the T_2_ relaxation time in the tumour ROI of Chit−IOCO−Cy5 became insignificantly different to the T_2_ at Day 0. This suggested that the non-specific uptake of Chit−IOCO−Cy5 observed at 24 h timepoint could have been washed off by the tumour interstitial fluids which were found in solid tumours and shown to hinder effective delivery to anti-cancer drugs [103,104]. Interestingly, imaging at Day 8 (5-day post second injection) revealed excellent retention of both Chit−IOCO−Cy5 and Chit−IOCO−MTX−Cy5 in the tumours. This could be due to the EPR effect that the Chit−IOCO-Cy5 remained in the interstitial fluids and blood pool gradually leaked into the tumours advantaging from the abnormal vascularisation in TME [105]. These findings could provide an explanation whereby in comparison to control group, all treated groups except free MTX group displayed a significant decrease in tumour size. The non-specific accumulation of Chit−IOCO−Cy5 at the tumour site along with the cancer killing effects of Chit−IOCO−Cy5 revealed in *in vitro* studies potentially led to significant inhibition of tumour progression. Overall, the enhanced uptake of Chit-IOCO-MTX-Cy5 in comparison to Chit-IOCO-Cy5 within the tumours as displayed via MRI and fluorescence imaging strongly corroborates our therapeutic studies performed in Fig. 6. The changes in tumours sizes that could be visualised on the MRI images also allow non-invasive and longitudinal measurements of treatment effects given that consistent acquisition protocols were applied.

## 5 Conclusion

In summary, this study reported the feasibility of Chit−IOCO−MTX-Cy5, a MTX labelled chitosan nanocomposite containing nanoceria as ROS regulating agent, iron oxide as MRI contrast agent and MTX as active targeting ligand and anti-cancer agent, as a nanoplatform for cancer theranostics. *In vitro* findings presented Chit−IOCO−MTX-Cy5 as a promising targeted cancer therapeutic agent with excellent MRI negative contrast efficacy and improved cytocompatibility of MTX. Chit−IOCO−MTX-Cy5 exhibited synergistic anti-cancer effect compared to MTX alone and Chit-IOCO-Cy5 *in vitro* via increasing intracellular ROS which resulted in apoptosis of U87MG cell. Incorporation of MTX to Chit−IOCO enhanced accumulation of NPs to tumour site within the first 3 days compared to Chit−IOCO as demonstrated by *in vivo* MR imaging. Both Chit−IOCO and Chit−IOCO−MTX-Cy5 demonstrated significant tumour inhibition *in vivo* in comparison to MTX which was an established chemotherapeutic agent. Nonetheless, Chit-IOCO-MTX-Cy5 displayed maximal tumour growth inhibition suggesting that MTX conjugated to Chit-IOCO synergistically enhanced anti-cancer abilities in comparison to free MTX and Chit-IOCO without MTX conjugation. Importantly, the anti-cancer effect of the developed nanocomposites was long-lasting and remained for many days after the treatment stopped, which was not observed in free MTX group. This finding highlights the great advantage of our nanocomposites compared to conventional chemotherapy based on MTX. Notably, biochemical and biosafety analysis of NPs *in vivo* established no significant toxicity. Overall, our nanohybrid NPs exhibited outstanding abilities in the concurrent utilisation for a safe, targeted and effective treatment, accompanied with real time monitoring via MRI in a U87MG model. Our chitosan nanocomposites with iron oxide and nanoceria, functionalised with MTX, holds great potential in achieving simultaneous, non-invasive monitoring and treatment of a wide range of ROS-related diseases.

## CRediT authorship contribution statement

**Joyce L.Y. Tang:** Writing – original draft, Visualization, Methodology, Investigation. **Shehzahdi S. Moonshi:** Writing – original draft, Visualization, Supervision, Methodology, Investigation. **Yuao Wu:** Writing – review & editing, Supervision, Methodology, Investigation. **Gary Cowin:** Methodology, Investigation. **Karla X. Vazquez- Prada:** Investigation. **Huong D.N. Tran:** Investigation. **Andrew C. Bulmer:** Methodology, Investigation. **Hang Thu Ta:** Writing – review & editing, Supervision, Resources, Project administration, Methodology, Funding acquisition, Conceptualization.

## Declaration of competing interest

The authors declare the following financial interests/personal relationships which may be considered as potential competing interests:Hang Thu Ta reports financial support was provided by 10.13039/501100000925National Health and Medical Research Council. Hang Thu Ta reports financial support was provided by 10.13039/501100001030National Heart Foundation of Australia. If there are other authors, they declare that they have no known competing financial interests or personal relationships that could have appeared to influence the work reported in this paper.

## Data Availability

Data will be made available on request.
