# Supplementary material for: A methotrexate labelled dual metal oxide nanocomposite for long-lasting anti-cancer theranostics
Source: Mater Today Bio. 2024 Dec 5;30:101377. doi: 10.1016/j.mtbio.2024.101377 (PMC11683249; doi:10.1016/j.mtbio.2024.101377)
Supplement: Multimedia component 1 [file mmc1.docx]

**Supporting Information**

**A METHOTREXATE LABELLED DUAL METAL OXIDE NANOCOMPOSITE FOR LONG-LASTING ANTI-CANCER THERANOSTICS**

Joyce Tang^1,2^†, Shehzahdi S. Moonshi^1,2^†, Yuao Wu^1,2^, Gary Cowin^4^, Karla X. Vazquez Prada^1,3^, Huong D.N. Tran^1,3^, Andrew C. Bulmer^5^, Hang Thu Ta^1,2,3*^

^1^ Queensland Micro- and Nanotechnology Centre, Griffith University, Nathan, Queensland 4111, Australia

^2^ School of Environment and Science, Griffith University, Nathan, Queensland 4111, Australia.

^3^Australian Institute for Bioengineering and Nanotechnology, University of Queensland, St Lucia, Queensland 4072, Australia

^4^ National Imaging Facility, Centre for Advanced Imaging, University of Queensland, St Lucia, Queensland 4072, Australia

^5^ School of Pharmacy and Medical Sciences, Griffith University, Southport, Queensland 4215, Australia

* Correspondence: Hang Thu Ta ([h.ta@griffith.edu.au](mailto:h.ta@griffith.edu.au))

† These authors contribute equally to this work.

**Corresponding author:**

**Hang Thu Ta**, BEng, MSc, PhD

**Professor**, School of Environment and Science, and Queensland Micro- and Nanotechnology, Griffith University, Nathan Campus, Brisbane QLD 4111, Australia

Office: +61 (7) 3735 5384

Email: [h.ta@griffith.edu.au](mailto:h.ta@griffith.edu.au)

Website: <https://hangta.group/>

<https://experts.griffith.edu.au/27034-hang-ta>


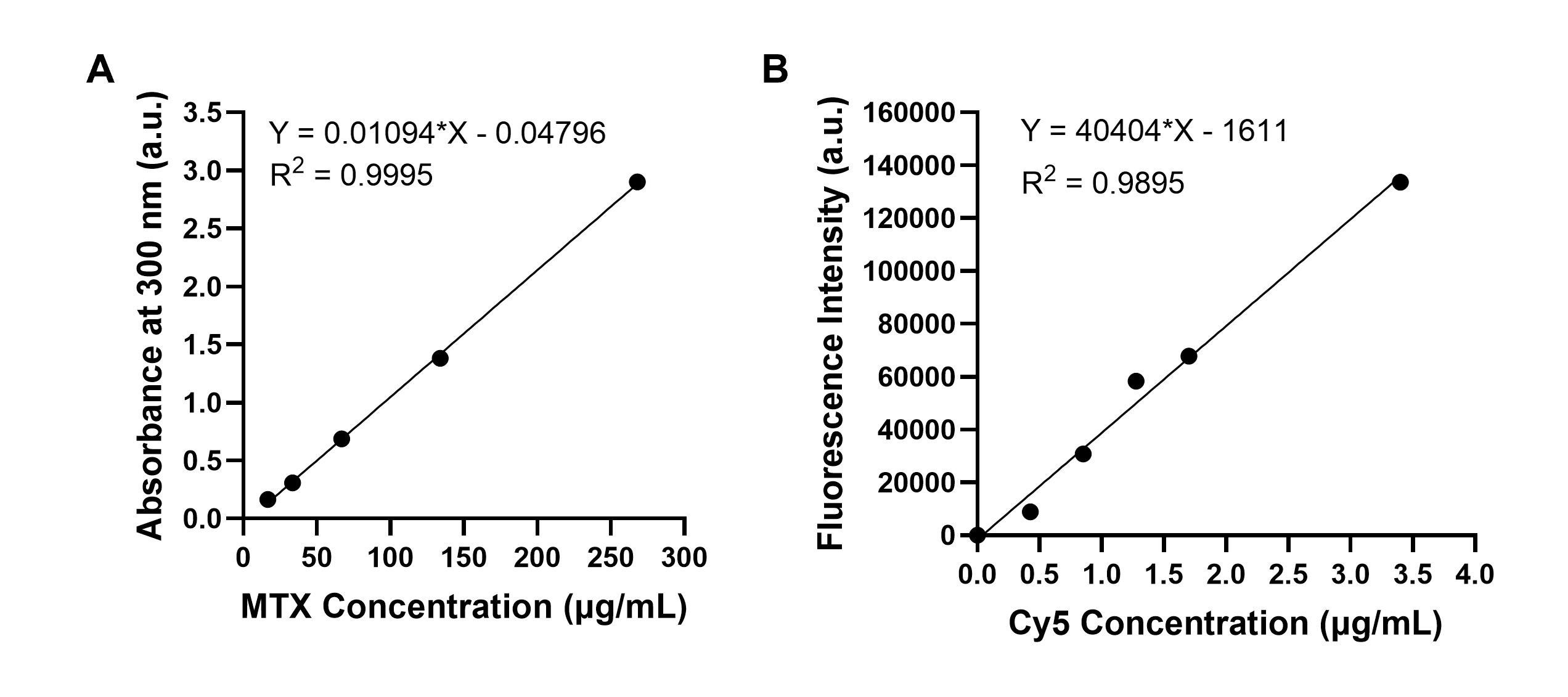


**Figure S1.** Calibration curves of methotrexate (MTX) and Cy5−COOH for determination of the amount of MTX and Cy5 conjugated to Chit−IOCO. **(A)** MTX standard curve generated from the absorbance readings of free MTX at different concentrations measured at 300 nm using CLARIOstar® Plus plate reader. **(B)** Cy5 standard curve plotted from the fluorescence intensities of free Cy5−COOH at different concentrations measured at 633/647 nm (excitation/emission) using CLARIOstar® Plus plate reader.


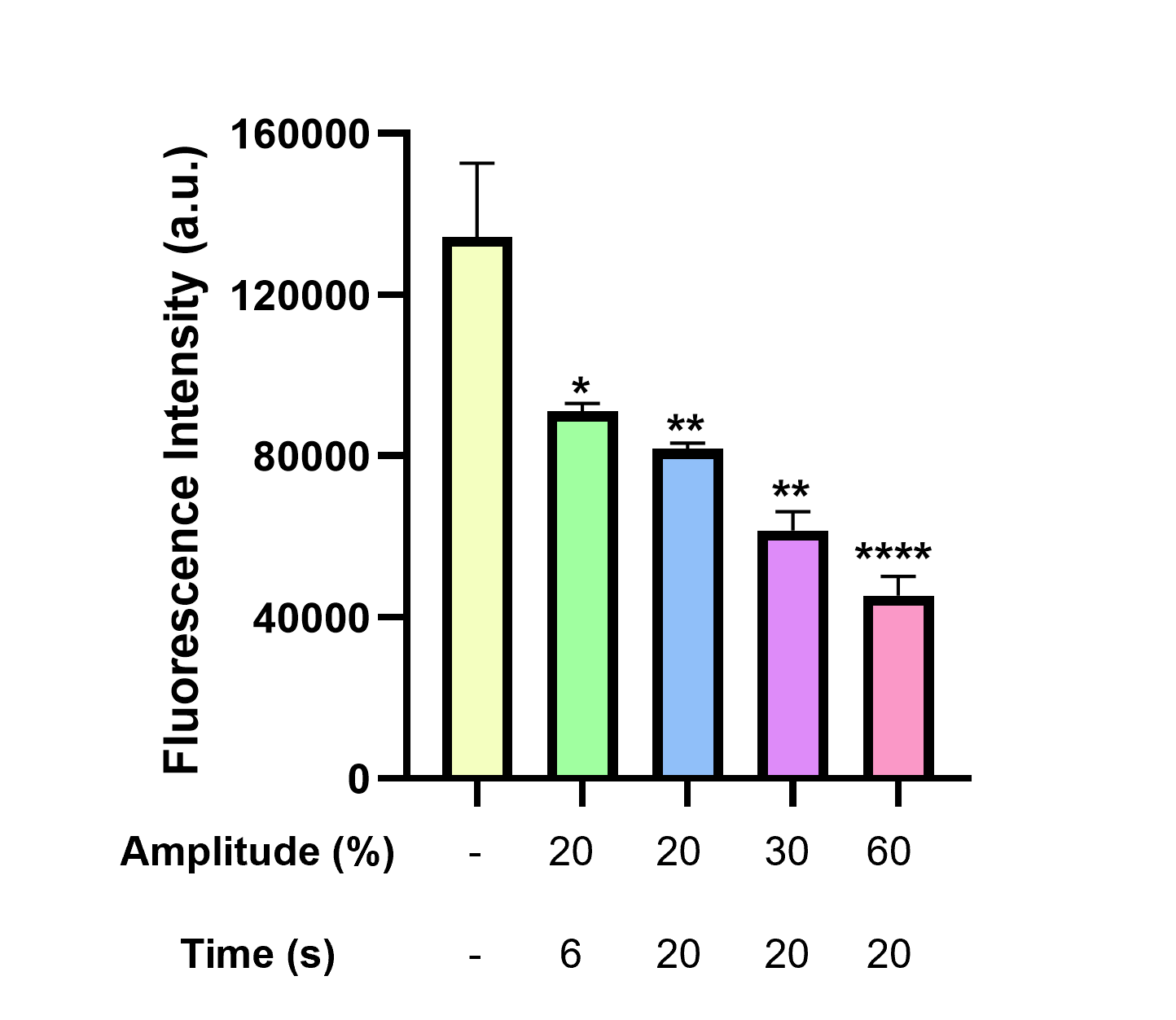


**Figure S2.** Effects of sonication on the fluorescence intensities of Cy5−COOH. Cy5-COOH solutions were sonicated at varying amplitude and time. Flluorescence intensities were then measured at 633/647 nm (excitation/emission) using CLARIOstar® Plus plate reader. *p ≤ 0.05, **p ≤ 0.01, ****p ≤ 0.0001, One-way ANOVA with Dunnett’s multiple comparisons test comparing control Cy5 solution (no sonication) to sonicated solutions.


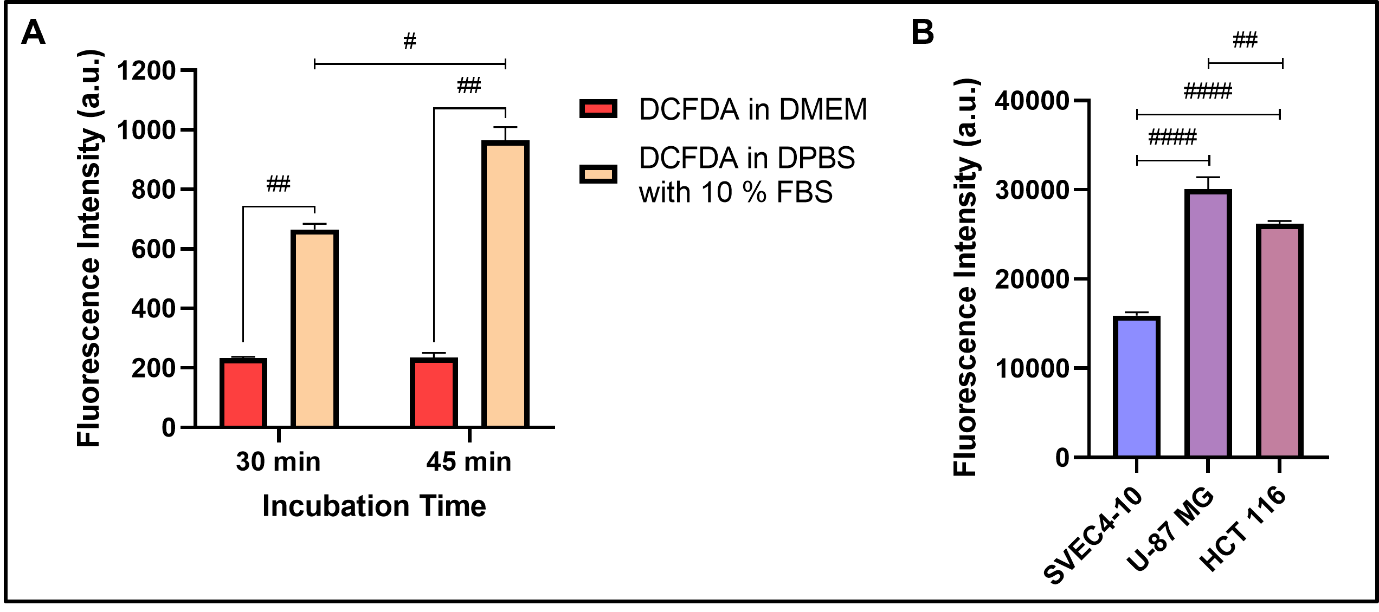


**Figure S3**. **DCFDA assays to determine changes in intracellular ROS.** (A) Optimisation of assay parameters. U-87 MG cells were seeded and stained with 25 μM DCFDA reagent in complete DMEM or in DPBS with 10 % FBS for 30- or 45-min at 37°C and 5 % CO2. Fluorescence readings were taken at 485/535 nm (excitation/emission) using CLARIOstar® Plus plate reader. (B) Differences in basal ROS levels between endothelial cell lines and cancer cell lines U-87MG and HCT 116.

**Table S1.** Half maximal inhibitory concentration IC_50_ of U-87 MG and HCT 116 cells treated with free MTX, Chit−IOCO and Chit−IOCO−MTX for 48 h. IC_50_ values were determined from the non-linear regression analyses of cell viability using GraphPad Prism.

| Cell Line | Treatment | MTX IC_50_ (µM) | Ce IC_50_ (µM) | Combination Index (CI) |
| --- | --- | --- | --- | --- |
| U-87 MG | **Free MTX** | 31.37 | - | 0.247 |
|  | **Chit−IOCO** | - | 92.90 |  |
|  | **Chit−IOCO−MTX** | 2.22 | 16.52 |  |
| HCT 116 | **Free MTX** | 5.34 | - | 0.497 |
|  | **Chit−IOCO** | - | 398.11 |  |
|  | **Chit−IOCO−MTX** | 2.49 | 12.25 |  |
